# Effects of PEEP on regional ventilation-perfusion mismatch in the acute respiratory distress syndrome

**DOI:** 10.1186/s13054-022-04085-y

**Published:** 2022-07-11

**Authors:** Bertrand Pavlovsky, Antonio Pesenti, Elena Spinelli, Gaetano Scaramuzzo, Ines Marongiu, Paola Tagliabue, Savino Spadaro, Giacomo Grasselli, Alain Mercat, Tommaso Mauri

**Affiliations:** 1 Department of Anesthesia, Critical Care and Emergency, IRCCS (Institute for Treatment and Research) Ca’ Granda Maggiore Policlinico Hospital Foundation, Via Sforza 35, 20122 Milan, Italy; 2 Vent’Lab, Medical Intensive Care Unit Department, Angers University Hospital, Angers, France; 3 Intensive Care Unit, Department of Morphology, Surgery and Experimental Medicine, Sant’Anna University Hospital, Ferrara, Italy; 4 Department of Pathophysiology and Transplantation, University of Milan, Milan, Italy; 5 Department of Medicine, University of Angers, Angers, France

**Keywords:** Acute lung injury, Electrical impedance tomography, Multiple inert gas elimination technique, Recruitment-on-inflation ratio, COVID-19

## Abstract

**Purpose:**

In the acute respiratory distress syndrome (ARDS), decreasing Ventilation-Perfusion $\left( {{{\dot{V}} \mathord{\left/ {\vphantom {{\dot{V}} {\dot{Q}}}} \right. \kern-\nulldelimiterspace} {\dot{Q}}}} \right)$ mismatch might enhance lung protection. We investigated the regional effects of higher Positive End Expiratory Pressure (PEEP) on ${{\dot{V}} \mathord{\left/ {\vphantom {{\dot{V}} {\dot{Q}}}} \right. \kern-\nulldelimiterspace} {\dot{Q}}}$ mismatch and their correlation with recruitability. We aimed to verify whether PEEP improves regional ${{\dot{V}} \mathord{\left/ {\vphantom {{\dot{V}} {\dot{Q}}}} \right. \kern-\nulldelimiterspace} {\dot{Q}}}$ mismatch, and to study the underlying mechanisms.

**Methods:**

In fifteen patients with moderate and severe ARDS, two PEEP levels (5 and 15 cmH_2_O) were applied in random order. ${{\dot{V}} \mathord{\left/ {\vphantom {{\dot{V}} {\dot{Q}}}} \right. \kern-\nulldelimiterspace} {\dot{Q}}}$ mismatch was assessed by Electrical Impedance Tomography at each PEEP. Percentage of ventilation and perfusion reaching different ranges of ${{\dot{V}} \mathord{\left/ {\vphantom {{\dot{V}} {\dot{Q}}}} \right. \kern-\nulldelimiterspace} {\dot{Q}}}$ ratios were analyzed in 3 gravitational lung regions, leading to precise assessment of their distribution throughout different ${{\dot{V}} \mathord{\left/ {\vphantom {{\dot{V}} {\dot{Q}}}} \right. \kern-\nulldelimiterspace} {\dot{Q}}}$ mismatch compartments. Recruitability between the two PEEP levels was measured by the recruitment-to-inflation ratio method.

**Results:**

In the non-dependent region, at higher PEEP, ventilation reaching the normal ${{\dot{V}} \mathord{\left/ {\vphantom {{\dot{V}} {\dot{Q}}}} \right. \kern-\nulldelimiterspace} {\dot{Q}}}$ compartment (*p* = 0.018) increased, while it decreased in the high ${{\dot{V}} \mathord{\left/ {\vphantom {{\dot{V}} {\dot{Q}}}} \right. \kern-\nulldelimiterspace} {\dot{Q}}}$ one (*p* = 0.023). In the middle region, at PEEP 15 cmH_2_O, ventilation and perfusion to the low ${{\dot{V}} \mathord{\left/ {\vphantom {{\dot{V}} {\dot{Q}}}} \right. \kern-\nulldelimiterspace} {\dot{Q}}}$ compartment decreased (*p* = 0.006 and *p* = 0.011) and perfusion to normal ${{\dot{V}} \mathord{\left/ {\vphantom {{\dot{V}} {\dot{Q}}}} \right. \kern-\nulldelimiterspace} {\dot{Q}}}$ increased (*p* = 0.003). In the dependent lung, the percentage of blood flowing through the non-ventilated compartment decreased (*p* = 0.041). Regional ${{\dot{V}} \mathord{\left/ {\vphantom {{\dot{V}} {\dot{Q}}}} \right. \kern-\nulldelimiterspace} {\dot{Q}}}$ mismatch improvement was correlated to lung recruitability and changes in regional tidal volume.

**Conclusions:**

In patients with ARDS, higher PEEP optimizes the distribution of both ventilation (in the non-dependent areas) and perfusion (in the middle and dependent lung). Bedside measure of recruitability is associated with improved ${{\dot{V}} \mathord{\left/ {\vphantom {{\dot{V}} {\dot{Q}}}} \right. \kern-\nulldelimiterspace} {\dot{Q}}}$ mismatch.

**Supplementary Information:**

The online version contains supplementary material available at 10.1186/s13054-022-04085-y.

## Introduction

The Acute Respiratory Distress Syndrome (ARDS) is characterized by hypoxemia despite positive airway pressure and by bilateral inflammatory infiltrates [1]. Pathophysiological mechanisms causing ARDS include: flooding of the alveolar space by edema and infiltration by inflammatory cells [2]; lung collapse due to superimposed weight [3]; small airway compression and edema due to cyclic re-opening [4]; diffuse micro-thrombosis [5]; direct vascular injury [6]. All these concur to generate pulmonary ventilation-perfusion (${{\dot{V}} \mathord{\left/ {\vphantom {{\dot{V}} {\dot{Q}}}} \right. \kern-\nulldelimiterspace} {\dot{Q}}}$) mismatch to highly variable and unpredictable extent [7]. Pulmonary angiography [8], volumetric capnography [9], pulmonary artery catheter [10], multiple inert gas elimination technique (MIGET) [11] and model-based measures of changes in oxygenation [12] all represent bedside methods to assess global ${{\dot{V}} \mathord{\left/ {\vphantom {{\dot{V}} {\dot{Q}}}} \right. \kern-\nulldelimiterspace} {\dot{Q}}}$ mismatch, which may be a sensitive *marker* of the overall severity of ARDS. Indeed, bedside estimates of global ${{\dot{V}} \mathord{\left/ {\vphantom {{\dot{V}} {\dot{Q}}}} \right. \kern-\nulldelimiterspace} {\dot{Q}}}$ mismatch are correlated with ARDS outcome [13, 14].

Pilot clinical and experimental data indicated that regional ${{\dot{V}} \mathord{\left/ {\vphantom {{\dot{V}} {\dot{Q}}}} \right. \kern-\nulldelimiterspace} {\dot{Q}}}$ mismatch may not simply be a marker of ARDS severity, but rather a direct *mechanism* for ventilator-induced lung injury (VILI) [15, 16]. Areas of wasted ventilation are associated with an increased risk of local hypocapnic injury [17] and of barotrauma in the remaining lung [18]; larger fraction of wasted perfusion is correlated with smaller size of the normally aerated lung, increasing the risk of regional volutrauma [18], and with local initiation of ischemic inflammatory lung injury [19]. Thus, bedside measures of regional ${{\dot{V}} \mathord{\left/ {\vphantom {{\dot{V}} {\dot{Q}}}} \right. \kern-\nulldelimiterspace} {\dot{Q}}}$ mismatch may disclose higher risk for VILI and guide personalized treatments aimed to limit this risk.

Electrical impedance tomography (EIT) is a non-irradiant, non-invasive bedside imaging monitor of ventilation and perfusion distribution [16, 20]. We recently used the bolus saline method during inspiratory breath-old to assess the global ${{\dot{V}} \mathord{\left/ {\vphantom {{\dot{V}} {\dot{Q}}}} \right. \kern-\nulldelimiterspace} {\dot{Q}}}$ mismatch by EIT in ARDS patients and we disclosed significant association with mortality [16]. For the present study, we developed a novel EIT-based analysis to quantify the regional fraction of perfusion and ventilation reaching compartments with different values of ${{\dot{V}} \mathord{\left/ {\vphantom {{\dot{V}} {\dot{Q}}}} \right. \kern-\nulldelimiterspace} {\dot{Q}}}$ ratios in 3 gravitational lung areas.

Positive End-Expiratory Pressure (PEEP) may have opposite effects on regional ${{\dot{V}} \mathord{\left/ {\vphantom {{\dot{V}} {\dot{Q}}}} \right. \kern-\nulldelimiterspace} {\dot{Q}}}$ mismatch, depending on its ability to recruit collapsed lung regions. When PEEP stabilizes recruitment, restoring airway patency and alveolar aeration [4], regional ${{\dot{V}} \mathord{\left/ {\vphantom {{\dot{V}} {\dot{Q}}}} \right. \kern-\nulldelimiterspace} {\dot{Q}}}$ mismatch might decrease, especially in the middle-dependent lung. At the opposite, the application of higher PEEP to non-recruitable lungs might worsen ${{\dot{V}} \mathord{\left/ {\vphantom {{\dot{V}} {\dot{Q}}}} \right. \kern-\nulldelimiterspace} {\dot{Q}}}$ mismatch by increasing both wasted ventilation (through overdistension) [18] and wasted perfusion (by redistribution of blood flow to collapsed areas) [21]. Animal studies confirmed that redistribution of ventilation towards dorsal lung regions may be a key mechanism for PEEP to improve regional ${{\dot{V}} \mathord{\left/ {\vphantom {{\dot{V}} {\dot{Q}}}} \right. \kern-\nulldelimiterspace} {\dot{Q}}}$ mismatch [22, 23]. To date, no study explored the regional effects of PEEP on ${{\dot{V}} \mathord{\left/ {\vphantom {{\dot{V}} {\dot{Q}}}} \right. \kern-\nulldelimiterspace} {\dot{Q}}}$ mismatch in ARDS patients.

In the present study, we assessed regional ${{\dot{V}} \mathord{\left/ {\vphantom {{\dot{V}} {\dot{Q}}}} \right. \kern-\nulldelimiterspace} {\dot{Q}}}$ mismatch by EIT at PEEP 5 and 15 cmH_2_O in moderate and severe ARDS patients. Study aim was to describe the mechanisms underlying regional effects of higher PEEP on ${{\dot{V}} \mathord{\left/ {\vphantom {{\dot{V}} {\dot{Q}}}} \right. \kern-\nulldelimiterspace} {\dot{Q}}}$ mismatch in patients with ARDS.

## Materials and methods

### Patients population

A convenience sample of fifteen intubated, sedated and paralyzed patients admitted to the general intensive care unit (ICU) of the Maggiore Policlinico Hospital in Milan, Italy, with moderate or severe ARDS [1] were enrolled in the study, within 7 days from intubation. Patients had to be older than 18, without pregnancy, and fulfill moderate or severe ARDS criteria according to the Berlin definition. They were enrolled in the study according to the investigators availability, without other pre-specified condition.

### Study protocol

Each patient was randomly assigned to a cross-over PEEP strategy: (1) PEEP 5 cmH_2_O and then 15 cmH_2_O, or (2) PEEP 15 cmH_2_O followed by 5 cmH_2_O. Each PEEP level was applied for 30 min. EIT-based ventilation and perfusion analysis, respiratory mechanics, arterial and central venous blood gases and hemodynamics were assessed at each step. Further details are available in the Online Supplement. Vasopressor doses remained constant during the study procedures.

The recruitment-to-inflation (*R/I*) ratio was computed between the two PEEP levels using EIT to quantify the change in end expiratory lung volume, as previously described [24].

### Ventilation and perfusion assessment

EIT-based quantification of regional ventilation and perfusion was performed as in previous studies [16, 24, 25]. A 10 ml bolus of NaCl 5% was infused during an end-inspiratory pause inducing an impedance drop, and perfusion distribution was measured by a first-pass dilution kinetic model [24]. Ventilation and perfusion maps were reconstructed offline by dedicated software (Dräger EIT Perfusion v.1.0, Dräger, Lubeck, Germany) [16, 24, 25].

### ***Regional analysis of ***${{\dot{V}} \mathord{\left/ {\vphantom {{\dot{V}} {\dot{Q}}}} \right. \kern-\nulldelimiterspace} {\dot{Q}}}$*** mismatch***

To enhance the understanding of ${{\dot{V}} \mathord{\left/ {\vphantom {{\dot{V}} {\dot{Q}}}} \right. \kern-\nulldelimiterspace} {\dot{Q}}}$ mismatch, we used a novel approach to quantify the regional distribution of ventilation and perfusion across units characterized by different values of ${{\dot{V}} \mathord{\left/ {\vphantom {{\dot{V}} {\dot{Q}}}} \right. \kern-\nulldelimiterspace} {\dot{Q}}}$ ratios. For this purpose, maps of the pixel-level relative distribution of ventilation and perfusion were analyzed by custom-made dedicated software (MATLAB, MathWorks, Natick, MA, USA).

${{\dot{V}} \mathord{\left/ {\vphantom {{\dot{V}} {\dot{Q}}}} \right. \kern-\nulldelimiterspace} {\dot{Q}}}$ distribution was evaluated within 3 regions of interest (ROI) of the total lung map, which was previously delineated by superimposition of the ventilation and perfusion maps. The 3 ROIs were obtained by dividing the ventro-dorsal axis of the lung map into 3 same-height parts, namely non-dependent, middle and dependent [26]. Thus, ventilation and perfusion fractions reaching each region were expressed as non-dependent, middle and dependent ventilation and perfusion ($\dot{V}$_ND_, $\dot{V}$_M_, $\dot{V}$_D_ and $\dot{Q}$_ND_, $\dot{Q}$_M_ and $\dot{Q}_{D}$, respectively).

The ${{\dot{V}} \mathord{\left/ {\vphantom {{\dot{V}} {\dot{Q}}}} \right. \kern-\nulldelimiterspace} {\dot{Q}}}$ ratio of each pixel within each region was transformed into a logarithmic value, and then rounded to its first decimal on a scale between − 1 (corresponding to a ${{\dot{V}} \mathord{\left/ {\vphantom {{\dot{V}} {\dot{Q}}}} \right. \kern-\nulldelimiterspace} {\dot{Q}}}$ ratio of 0.1) and 1 (${{\dot{V}} \mathord{\left/ {\vphantom {{\dot{V}} {\dot{Q}}}} \right. \kern-\nulldelimiterspace} {\dot{Q}}}$ ratio of 10). Then, the logarithmic ${{\dot{V}} \mathord{\left/ {\vphantom {{\dot{V}} {\dot{Q}}}} \right. \kern-\nulldelimiterspace} {\dot{Q}}}$ ratios of all pixels were grouped to obtain 21 discrete ranges, by rounding their log(${{\dot{V}} \mathord{\left/ {\vphantom {{\dot{V}} {\dot{Q}}}} \right. \kern-\nulldelimiterspace} {\dot{Q}}}$) value to the nearest 0.1 decimal number. The distribution curves of ventilation and perfusion fractions within these 21 log(${{\dot{V}} \mathord{\left/ {\vphantom {{\dot{V}} {\dot{Q}}}} \right. \kern-\nulldelimiterspace} {\dot{Q}}}$) ratio ranges were built for each patient in each region at each PEEP level (6 regional curves with 21 points per curve per patient, Additional file 1: Fig. S1 of the Online Supplement). More detailed information on the methods are available in the Additional file 1.

To improve visual understanding of the study results, we also built average curves of the distribution of ventilation and perfusion in the whole population at each PEEP level, by using the mean values for each log(${{\dot{V}} \mathord{\left/ {\vphantom {{\dot{V}} {\dot{Q}}}} \right. \kern-\nulldelimiterspace} {\dot{Q}}}$) compartment.

### ***Quantitative assessment of regional ***${{\dot{V}} \mathord{\left/ {\vphantom {{\dot{V}} {\dot{Q}}}} \right. \kern-\nulldelimiterspace} {\dot{Q}}}$*** mismatch***

Using the curves described above, a five-compartment model was first designed to assess the fraction of ventilation and perfusion reaching: (1) non-ventilated perfused units (${{\dot{V}} \mathord{\left/ {\vphantom {{\dot{V}} {\dot{Q}}}} \right. \kern-\nulldelimiterspace} {\dot{Q}}}$ ratio ≤ 0.1); (2) units with low ${{\dot{V}} \mathord{\left/ {\vphantom {{\dot{V}} {\dot{Q}}}} \right. \kern-\nulldelimiterspace} {\dot{Q}}}$ ratio (${{\dot{V}} \mathord{\left/ {\vphantom {{\dot{V}} {\dot{Q}}}} \right. \kern-\nulldelimiterspace} {\dot{Q}}}$ ratio 0.1–0.8); (3) units with normal ${{\dot{V}} \mathord{\left/ {\vphantom {{\dot{V}} {\dot{Q}}}} \right. \kern-\nulldelimiterspace} {\dot{Q}}}$ ratio (${{\dot{V}} \mathord{\left/ {\vphantom {{\dot{V}} {\dot{Q}}}} \right. \kern-\nulldelimiterspace} {\dot{Q}}}$ ratio 0.8–1.25); (4) units with high ${{\dot{V}} \mathord{\left/ {\vphantom {{\dot{V}} {\dot{Q}}}} \right. \kern-\nulldelimiterspace} {\dot{Q}}}$ ratio (${{\dot{V}} \mathord{\left/ {\vphantom {{\dot{V}} {\dot{Q}}}} \right. \kern-\nulldelimiterspace} {\dot{Q}}}$ ratio 1.25–10); (5) non-perfused ventilated units (${{\dot{V}} \mathord{\left/ {\vphantom {{\dot{V}} {\dot{Q}}}} \right. \kern-\nulldelimiterspace} {\dot{Q}}}$ ratio ≥ 10). The analysis was repeated for each lung region in all patients.

Then, the shape of the regional ventilation and perfusion over ${{\dot{V}} \mathord{\left/ {\vphantom {{\dot{V}} {\dot{Q}}}} \right. \kern-\nulldelimiterspace} {\dot{Q}}}$ ratios curves was also analyzed in a “MIGET-like” way, to provide their average distribution in terms of ${{\dot{V}} \mathord{\left/ {\vphantom {{\dot{V}} {\dot{Q}}}} \right. \kern-\nulldelimiterspace} {\dot{Q}}}$ ratio (Mean $\dot{V}$ and Mean $\dot{Q}$) and their skewness by logarithmic standard derivation (logSD$_{{\dot{V}}}$ and logSD$_{{\dot{Q}}}$), as previously described (see also the Online Supplement) [27].

Finally, precise assessment of regional wasted perfusion and wasted ventilation was calculated by the Eqs. (1) and (2).

1 $${\text{Wasted perfusion}} = \mathop \sum \limits_{i = 1}^{n} \left( {\log \left( {\frac{{\mathop {\dot{V}}\limits^{ \cdot } }}{{\dot{Q}}}} \right)_{i} *\dot{Q}_{i} } \right)$$

where n is the number of pixels in the functional EIT image within each ROI, including only units with ${{\dot{V}} \mathord{\left/ {\vphantom {{\dot{V}} {\dot{Q}}}} \right. \kern-\nulldelimiterspace} {\dot{Q}}}$ ratio < 1; please note that the log(${{\dot{V}} \mathord{\left/ {\vphantom {{\dot{V}} {\dot{Q}}}} \right. \kern-\nulldelimiterspace} {\dot{Q}}}$) ratio was considered as absolute value with no sign for this calculation.

2 $${\text{Wasted ventilation}} = \mathop \sum \limits_{i = 1}^{n} \left( {\log \left( {\frac{{\mathop {\dot{V}}\limits^{ \cdot } }}{{\dot{Q}_{i} }}} \right)_{i} *\dot{V}_{i} } \right)$$

where n is the number of pixels in the functional EIT image within each ROI, including only units with ${{\dot{V}} \mathord{\left/ {\vphantom {{\dot{V}} {\dot{Q}}}} \right. \kern-\nulldelimiterspace} {\dot{Q}}}$ ratio > 1; please note that the log(${{\dot{V}} \mathord{\left/ {\vphantom {{\dot{V}} {\dot{Q}}}} \right. \kern-\nulldelimiterspace} {\dot{Q}}}$) ratio was considered as absolute value with no sign for this calculation.

### Statistical analysis

Based on our experience, and similarly to other similar studies on this research field [20, 28], we selected a sample size of 15 patients to show physiologically relevant differences in regional ${{\dot{V}} \mathord{\left/ {\vphantom {{\dot{V}} {\dot{Q}}}} \right. \kern-\nulldelimiterspace} {\dot{Q}}}$ matching.

Results are expressed by median [25–75th quartiles] for quantitative data, and as number (percentage) for qualitative data.

Comparisons between variables assessed during the two study steps (e.g. PEEP 5 vs 15 cmH_2_O) were performed by paired t-test or Wilcoxon test, as appropriate based on gaussian distribution test (Shapiro–Wilk). Correlations were assessed by linear regression model.

A two-tailed *p*-value below 0.05 was considered as statistically significant. All statistical analyses were performed with Prism (GraphPad Prism v.9.2, La Jolla, CA, USA).

## Results

### Study population

We enrolled 15 patients with moderate and severe ARDS: main characteristics are described in Table 1. Median age was 60 [48–68] years, BMI was slightly elevated (27.3 [25.7–35.4] kg.m^−2^). SAPS II score at ICU admission was 40 [29–53], SOFA score on the day of the study was 6 [3–8] and number of extra-pulmonary organs failure was 1 [0–2]. ICU length of stay was 12 [7–25] days and hospital mortality was 40% (Table 1).

**Table 1:** Patient’s characteristics

	All patients *n* = 15
Demographics	
Age, years	60 [48–68]
Male gender (%)	10 (67)
Body Mass Index, kg.m^−2^	27.3 [25.7–35.4]
Comorbidities (%)	
Hypertension	7 (47)
Diabetes mellitus	6 (40)
Immunosuppression	2 (13)
Disease severity	
SAPS II at admission to the ICU	40 [29–53]
SOFA score at enrollment	6 [3–8]
ARDS etiology (%)	
COVID-19 pneumonia	9 (60)
Bacterial Pneumonia	4 (27)
Septic shock	2 (13)
Days from intubation	2 [2–5]
ICU length of stay, days	12 [17–25]
28-day mortality, n (%)	6 (40)
Clinical settings and gas exchange at enrollment	
PEEP, cmH_2_O	12 [10–12]
Tidal volume, mL.kg^−1^ PBW	6.7 [6.0–7.5]
RR, min^−1^	22 [16–26]
FiO_2_, %	60 [40–60]
PaO_2_/FiO_2_, mmHg	136 [106–188]
PaCO_2_, mmHg	44.9 [41.4–51.4]
pH	7.40 [7.36–7.46]

*ARDS* Acute respiratory distress syndrome, *COVID-19* Coronavirus disease 2019, *FiO*_*2*_ Inspired fraction of dioxygen, *ICU* Intensive Care Unit, *PaCO*_*2*_ Arterial partial pressure on carbon dioxide, *PaO*_*2*_ Arterial partial pressure on dioxygen, *PBW* Predicted body weight, *PEEP* Positive end expiratory pressure, *RR* Respiratory rate, *SOFA* Sequential organ failure assessment

ARDS etiology was COVID-19 for 9 (60%), bacterial pneumonia in 4 (27%) and septic shock in 2 patients (13%) (Table 1).

On the day of the study, patients were intubated since 2 [2–5] days and undergoing controlled mechanical ventilation with the following settings: PEEP 12 [10–12] cmH_2_O, tidal volume 6.7 [6.0–7.5] mL.kg^−1^ PBW, and respiratory rate 22 [16–26] min^−1^. PaO_2_/FiO_2_ was 136 [106–188] mmHg and PaCO_2_ 44.9 [41.4–51.4] mmHg (Table 1).

The order of PEEP levels application was randomized; 8 patients were studied first at 5, then 15 cmH2O, and 7 with a 15–5 sequence.

### Effects of higher PEEP on regional ${{\dot{V}} \mathord{\left/ {\vphantom {{\dot{V}} {\dot{Q}}}} \right. \kern-\nulldelimiterspace} {\dot{Q}}}$ mismatch

Regional changes in ventilation and perfusion are summarized in the Additional file 1: Table S1 of the Online Supplement. Of note, there was no significant difference in changes of ventilation or perfusion distribution between the two randomization order groups (Additional file 1: Table S2).

**Fig. 1:**
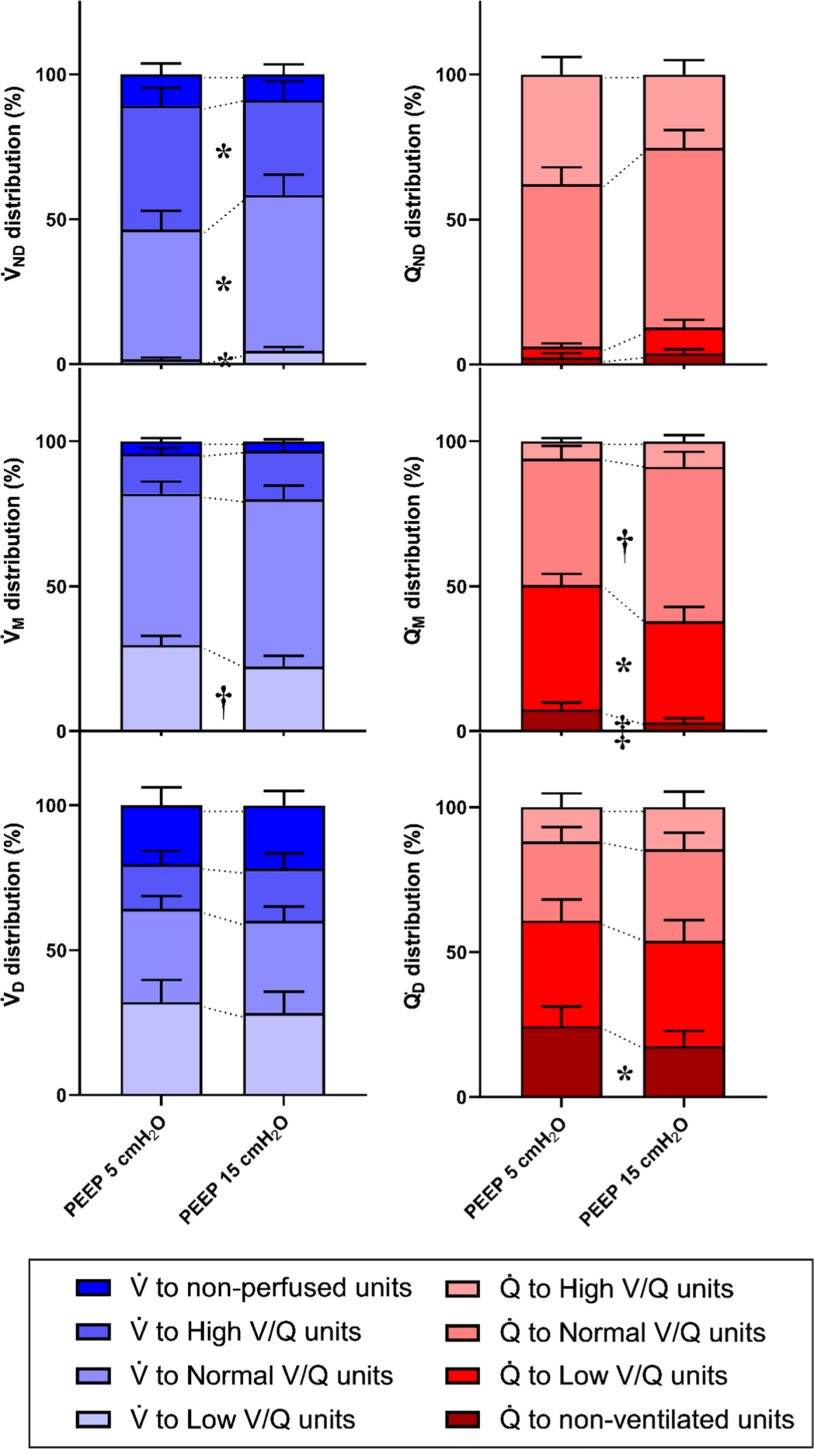
Regional distribution of ventilation ($\dot{V}$, blue bars) and perfusion ($\dot{Q}$, red bars) across areas with different ranges of ${{\dot{V}} \mathord{\left/ {\vphantom {{\dot{V}} {\dot{Q}}}} \right. \kern-\nulldelimiterspace} {\dot{Q}}}$ ratio at Positive End Expiratory Pressure (PEEP) 5 and 15 cmH_2_O. Histograms represent mean values (± SEM). ND: non-dependent part of the lungs, M: middle part of the lungs, D: dependent part of the lungs. *p < 0.05, †*p* < 0.01, ‡*p* < 0.001 by paired t-test

*In the non-dependent region*, higher PEEP induced a large decrease in the percentage of $\dot{V}$_ND_ reaching units with high ${{\dot{V}} \mathord{\left/ {\vphantom {{\dot{V}} {\dot{Q}}}} \right. \kern-\nulldelimiterspace} {\dot{Q}}}$ ratio (30 [7–47] vs. 46 [21–62] % of $\dot{V}$_ND_, *p* = 0.023) and the fraction of $\dot{V}$_ND_ reaching units with normal ${{\dot{V}} \mathord{\left/ {\vphantom {{\dot{V}} {\dot{Q}}}} \right. \kern-\nulldelimiterspace} {\dot{Q}}}$ increased (52 [32–89] vs. 42 [18–60] % of $\dot{V}$_ND_, *p* = 0.018). The non-dependent lung region suffered the only slight worsening of regional ${{\dot{V}} \mathord{\left/ {\vphantom {{\dot{V}} {\dot{Q}}}} \right. \kern-\nulldelimiterspace} {\dot{Q}}}$ mismatch: namely, an increased fraction of regional $\dot{V}$_ND_ to low ${{\dot{V}} \mathord{\left/ {\vphantom {{\dot{V}} {\dot{Q}}}} \right. \kern-\nulldelimiterspace} {\dot{Q}}}$ units (4 [0–10] vs. 0 [0–4] % of $\dot{V}$_ND_, *p* = 0.020) (Fig. 1). Re-distribution of perfusion by higher PEEP in non-dependent regions showed only slight modifications with similar trends: decrease in high ${{\dot{V}} \mathord{\left/ {\vphantom {{\dot{V}} {\dot{Q}}}} \right. \kern-\nulldelimiterspace} {\dot{Q}}}$ units and increase in normal ${{\dot{V}} \mathord{\left/ {\vphantom {{\dot{V}} {\dot{Q}}}} \right. \kern-\nulldelimiterspace} {\dot{Q}}}$ units (Fig. 1).

**Fig. 2:**
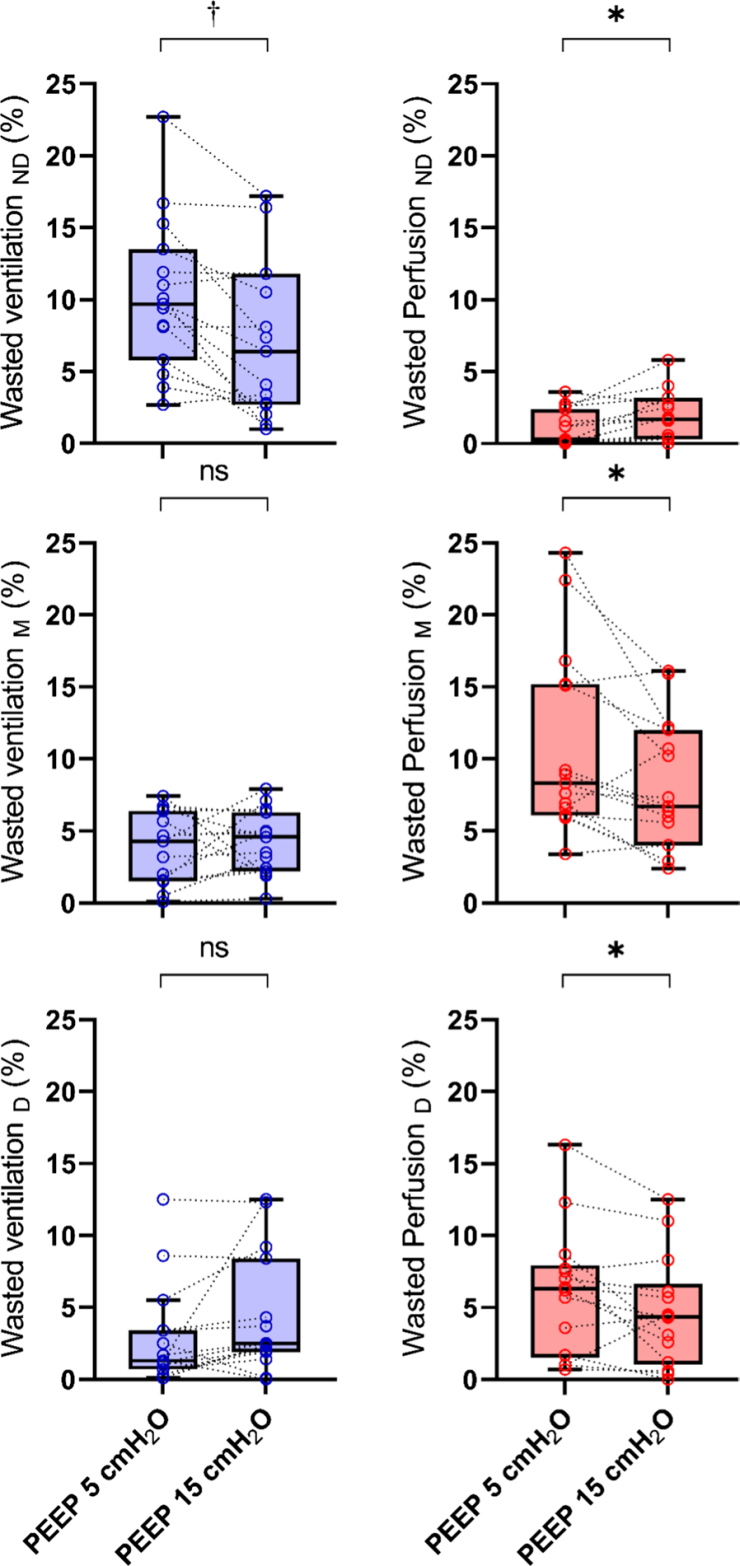
Regional fraction of wasted ventilation (blue boxes) and wasted perfusion (red boxes) at PEEP 5 and 15 cmH_2_O. Results are expressed in mean and Tukey box plots. ND: non-dependent part of the lungs, M: middle part of the lungs, D: dependent part of the lungs. *p < 0.05, †*p* < 0.01 by paired t-test

Within the non-dependent region, fraction of wasted ventilation decreased (6.4 [2.7–11.8] vs. 9.7 [5.8–13.5] % of $\dot{V}$_ND_, *p* = 0.002), and wasted perfusion slightly increased (1.7 [0.3–3.2] vs. 0.3 [0.1–2.4] % of $\dot{Q}$_ND_, *p* = 0.015) (Fig. 2).

*In the middle region of the lungs*, the percentage of ventilation to low ${{\dot{V}} \mathord{\left/ {\vphantom {{\dot{V}} {\dot{Q}}}} \right. \kern-\nulldelimiterspace} {\dot{Q}}}$ units decreased at PEEP 15 cmH_2_O (23 [8–30] vs. 29 [22–39] % of $\dot{V}$_M_, *p* = 0.006), and the $\dot{V}$_M_ reaching units with normal ${{\dot{V}} \mathord{\left/ {\vphantom {{\dot{V}} {\dot{Q}}}} \right. \kern-\nulldelimiterspace} {\dot{Q}}}$ increased (54 [46–79] vs. 50 [42–62] % of $\dot{V}$_M_, *p* = 0.151) (Fig. 1). In this region, the amount of perfusion reaching low ${{\dot{V}} \mathord{\left/ {\vphantom {{\dot{V}} {\dot{Q}}}} \right. \kern-\nulldelimiterspace} {\dot{Q}}}$ and non-ventilated units decreased at higher PEEP (42 [15–49] vs. 46 [32–54] % of $\dot{Q}$_*M*_, *p* = 0.011; and 1 [0–2] vs. 4 [1–12] % of $\dot{Q}$_*M*_, *p* < 0.001, respectively). Thus, perfusion flowing to normal ${{\dot{V}} \mathord{\left/ {\vphantom {{\dot{V}} {\dot{Q}}}} \right. \kern-\nulldelimiterspace} {\dot{Q}}}$ units became larger (50 [36–72] vs. 42 [31–56]% of $\dot{Q}$_*M*_, *p* = 0.003). (Fig. 1).

These changes led to a decrease in wasted perfusion in the middle lung region (6.7 [4.0–12.0] vs. 8.3 [6.1–15.2] % of $\dot{Q}$_*M*_, *p* = 0.014) (Fig. 2).

*In the dependent region*, ventilation distribution ($\dot{V_D}$) was only slightly modified by higher PEEP, while improvement in ${{\dot{V}} \mathord{\left/ {\vphantom {{\dot{V}} {\dot{Q}}}} \right. \kern-\nulldelimiterspace} {\dot{Q}}}$ matching consisted in a large decrease in the fraction of perfusion reaching non-ventilated units (8 [3–32] vs. 16 [8–26] % of $\dot{Q}$_*D*_, *p* = 0.041) (Fig. 1).

Regional wasted perfusion in the dependent lung decreased (4.4 [1.1–6.7] vs. 6.3 [1.5–8.0] % of $\dot{Q_D}$*, p *= 0.049) (Fig. 2).

**Fig. 3:**
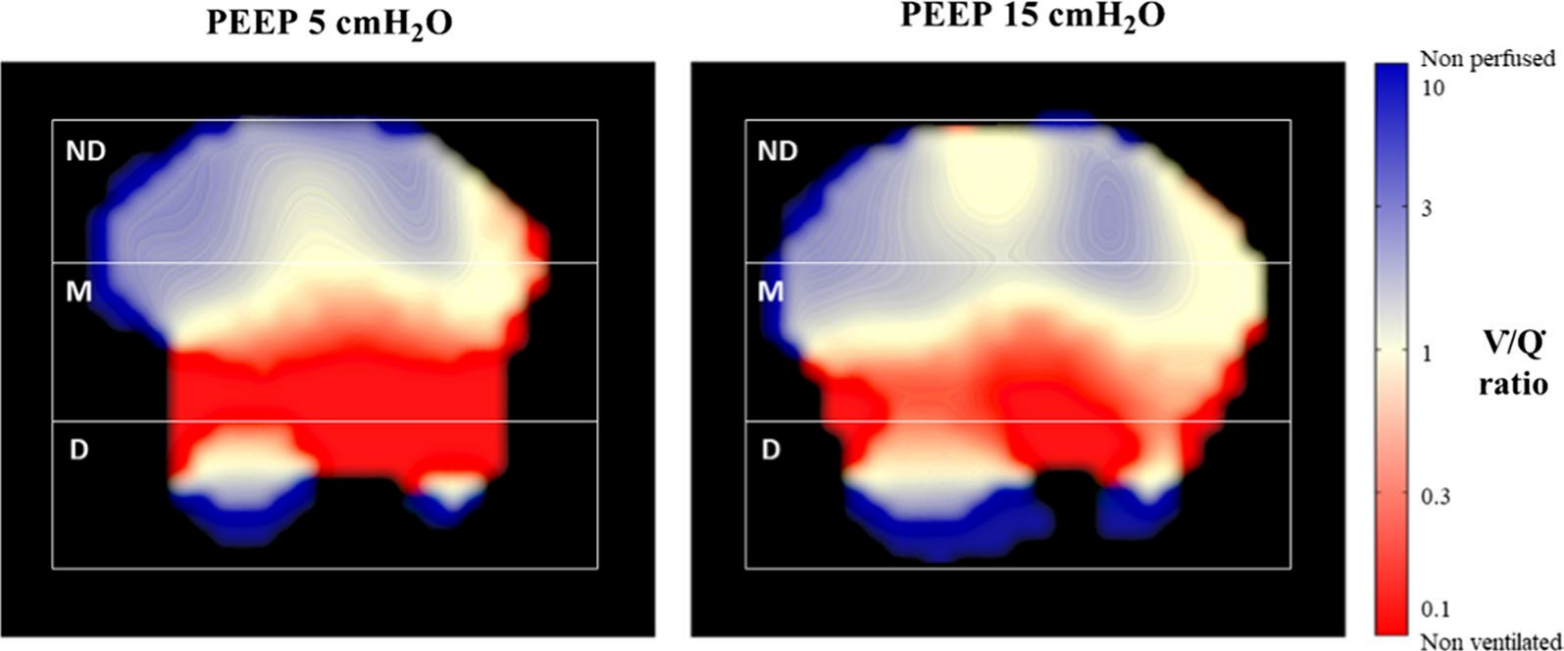
Topographic distribution of lung units with different Ventilation-Perfusion (${{\dot{V}} \mathord{\left/ {\vphantom {{\dot{V}} {\dot{Q}}}} \right. \kern-\nulldelimiterspace} {\dot{Q}}}$) ratios in a representative study patient at PEEP 5 and 15 cmH_2_O. ${{\dot{V}} \mathord{\left/ {\vphantom {{\dot{V}} {\dot{Q}}}} \right. \kern-\nulldelimiterspace} {\dot{Q}}}$ ratio was calculated as the pixel-level ventilation divided by perfusion measured by electrical impedance tomography. ${{\dot{V}} \mathord{\left/ {\vphantom {{\dot{V}} {\dot{Q}}}} \right. \kern-\nulldelimiterspace} {\dot{Q}}}$ ratio ranges from < 0.1 (non-ventilated units, red) to 1 (normal units, white) to > 10 (non-perfused units, blue). The color scale is displayed on the right side of the figure. ND: non-dependent part of the lungs, M: middle part of the lungs, D: dependent part of the lungs

Figure 3 shows the topographic distribution of ${{\dot{V}} \mathord{\left/ {\vphantom {{\dot{V}} {\dot{Q}}}} \right. \kern-\nulldelimiterspace} {\dot{Q}}}$ matching in a representative patient, with larger fraction of normal ${{\dot{V}} \mathord{\left/ {\vphantom {{\dot{V}} {\dot{Q}}}} \right. \kern-\nulldelimiterspace} {\dot{Q}}}$ units (white) and fewer non-ventilated (blue) and non-perfused (red) units across all lung regions at higher PEEP.

### Mechanisms underlying improvement of regional ${{\dot{V}} \mathord{\left/ {\vphantom {{\dot{V}} {\dot{Q}}}} \right. \kern-\nulldelimiterspace} {\dot{Q}}}$ mismatch

**Fig. 4:**
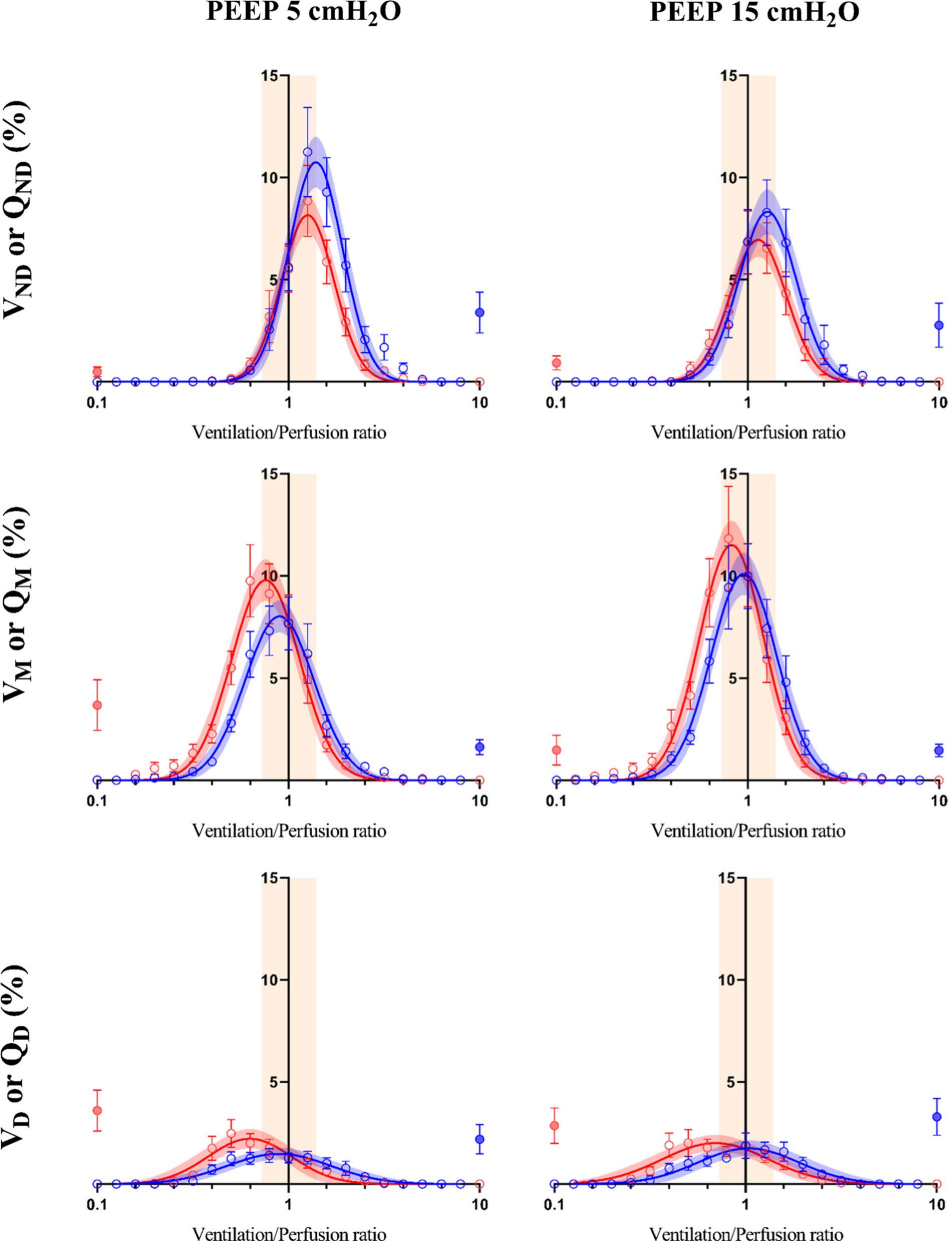
Regional distribution of the fraction of ventilation (blue) and perfusion (red) across all ${{\dot{V}} \mathord{\left/ {\vphantom {{\dot{V}} {\dot{Q}}}} \right. \kern-\nulldelimiterspace} {\dot{Q}}}$ ratios in the whole study population at PEEP 5 and 15 cmH_2_O. Mean (± SEM) values are expressed by open circles and whiskers. The solid lines correspond to the best fitting curves (with 95%CI). Fractions of perfusion to non-ventilated units and of ventilation to non-perfused areas are depicted with solid circles (red and blue, respectively). Range of normal ${{\dot{V}} \mathord{\left/ {\vphantom {{\dot{V}} {\dot{Q}}}} \right. \kern-\nulldelimiterspace} {\dot{Q}}}$ ratios (i.e., 0.8–1.25) is represented by the yellow box. ND: non-dependent part of the lungs, M: middle part of the lungs, D: dependent part of the lungs

Figure 4 graphically shows that, in all regions, the bell-shaped curves of the fraction of ventilation and perfusion plotted against the ${{\dot{V}} \mathord{\left/ {\vphantom {{\dot{V}} {\dot{Q}}}} \right. \kern-\nulldelimiterspace} {\dot{Q}}}$ ratios became more superimposed and with the central apex closer to the normal value of 1. Table 2 quantifies these changes in ventilation and perfusion distribution along the curves of ${{\dot{V}} \mathord{\left/ {\vphantom {{\dot{V}} {\dot{Q}}}} \right. \kern-\nulldelimiterspace} {\dot{Q}}}$ ratios, by presenting mean values (Mean $\dot{V}$ and Mean $\dot{Q}$, respectively) and their heterogeneity (logSD$_{{\dot{V}}}$ and logSD$_{{\dot{Q}}}$, respectively).

**Table 2:** Effect of positive end expiratory pressure (PEEP) on the distribution of regional Ventilation and Perfusion (cfr. Fig. 4 curves)

$\dot{V}$ and $\dot{Q}$ distribution	PEEP 5 cmH_2_O *n* = 15	PEEP 15 cmH_2_O *n* = 15	*p* value
*Non-dependent region*			
Mean $\dot{V}$	1.63 [1.27–2.39]	1.50 [1.16–1.77]	**0.040**
logSD$_{{\dot{V}}}$	0.05 [0.01–0.11]	0.04 [0.01–0.11]	0.991
Mean $\dot{Q}$	1.33 [1.08–1.48]	1.00 [0.93–1.26]	**0.009**
logSD$_{{\dot{Q}}}$	0.02 [0.01–0.05]	0.04 [0.01–0.102]	0.138
*Middle region*			
Mean $\dot{V}$	1.02 [0.85–1.14]	1.07 [0.91–1.13]	0.527
logSD$_{{\dot{V}}}$	0.08 [0.03–0.11]	0.06 [0.02–0.10]	0.157
Mean $\dot{Q}$	0.66 [0.52–0.79]	0.76 [0.66–0.87]	**0.001**
logSD$_{{\dot{Q}}}$	0.05 [0.03–0.12]	0.03 [0.02–0.09]	**0.021**
*Dependent region*			
Mean $\dot{V}$	1.25 [0.83–1.94]	1.28 [0.94–2.63]	0.978
logSD$_{{\dot{V}}}$	0.12 [0.08–0.19]	0.13 [0.11–0.25]	0.324
Mean $\dot{Q}$	0.46 [0.36–0.84]	0.50 [0.33–0.79]	0.131
logSD$_{{\dot{Q}}}$	0.11 [0.08–0.15]	0.09 [0.05–0.14]	0.202

Bold identifies significant differences

Mean $\dot{V}$ and Mean $\dot{Q}$ represent the mean ${{\dot{V}} \mathord{\left/ {\vphantom {{\dot{V}} {\dot{Q}}}} \right. \kern-\nulldelimiterspace} {\dot{Q}}}$ ratio of the ventilation and perfusion ${{\dot{V}} \mathord{\left/ {\vphantom {{\dot{V}} {\dot{Q}}}} \right. \kern-\nulldelimiterspace} {\dot{Q}}}$ distribution curves. LogSD$_{{\dot{V}}}$ and logSD_Q_ refers to the logarithm of their standard derivation, which is a marker of the curves skewness

*In the non-dependent regions*, both ventilation and perfusion were redistributed by higher PEEP and led to improved matching: indeed, Mean $\dot{V}$ and Mean $\dot{Q}$ decreased, becoming closer to 1 (Table 2).

*In the middle region of the lungs*, only distribution of perfusion was affected by higher PEEP, as Mean $\dot{Q}$ increased and became more similar to 1, while heterogeneity assessed by logSD$_{{\dot{Q}}}$ decreased (Table 2).

*In the dependent lung*, the indexes describing distribution of perfusion and ventilation reaching different ${{\dot{V}} \mathord{\left/ {\vphantom {{\dot{V}} {\dot{Q}}}} \right. \kern-\nulldelimiterspace} {\dot{Q}}}$ ratios didn’t change significantly by changing PEEP, and the above-mentioned improvements of ${{\dot{V}} \mathord{\left/ {\vphantom {{\dot{V}} {\dot{Q}}}} \right. \kern-\nulldelimiterspace} {\dot{Q}}}$ mismatch were likely due to small changes of both ventilation and perfusion distribution and homogeneity (Table 2).

Table S1 in the Online Supplement shows the percentage of ventilation and perfusion in the 3 regions considered as anatomical compartments: $\dot{V}$ decreased in the non-dependent zones, to reach the middle and dependent regions, while the distribution of perfusion between anatomical regions was barely affected.

### Correlation between improved ${{\dot{V}} \mathord{\left/ {\vphantom {{\dot{V}} {\dot{Q}}}} \right. \kern-\nulldelimiterspace} {\dot{Q}}}$ mismatch and recruitment

**Fig. 5:**
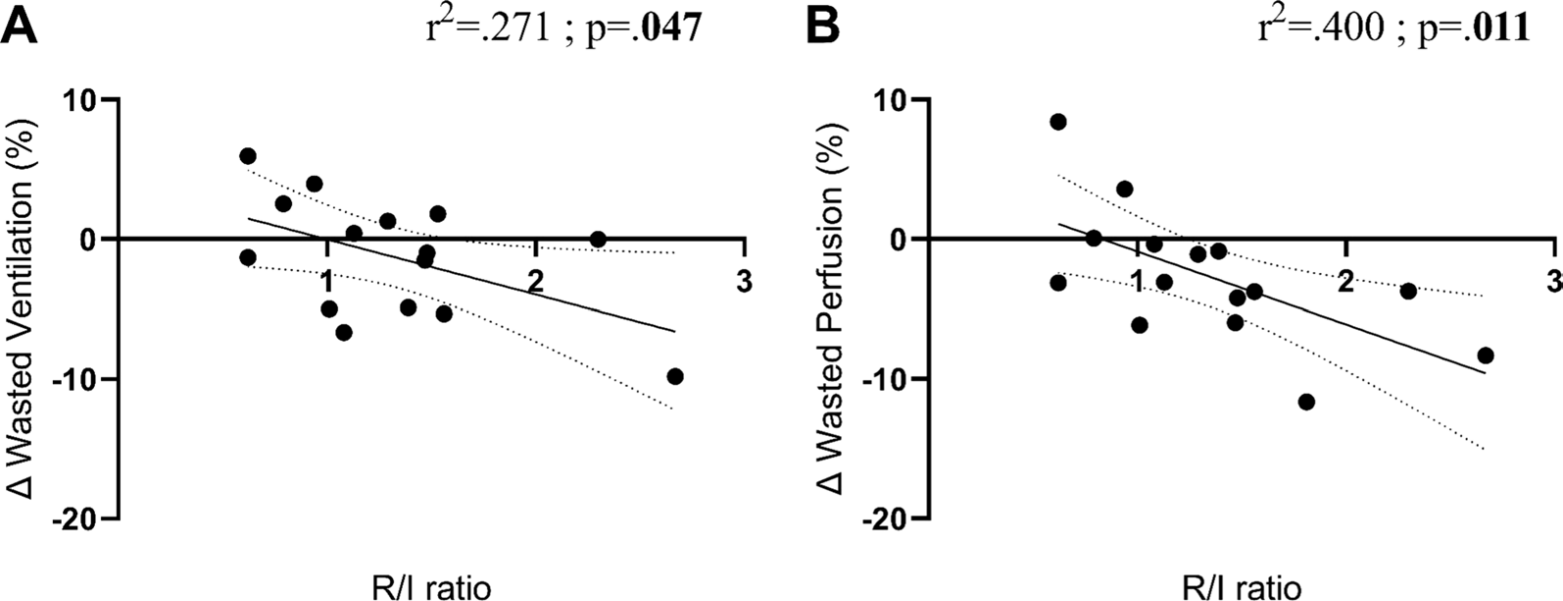
Correlations between recruitability (R/I ratio) and the improvement in Wasted Ventilation (**A**) and Wasted Perfusion (**B**) between PEEP 5 and 15 cmH_2_O

Median EIT-based R/I ratio measured between PEEP 15 and 5 cmH_2_O was 1.29 with high variability [IQR 1.01–1.53; range 0.62–2.67]. There was a correlation between the R/I ratio and the improvement in wasted ventilation induced by higher PEEP (*r*^2^ = 0.271, *p* = 0.047, Fig. 5A) and, more significantly, between the R/I ratio and the decrease in wasted perfusion (*r*^2^ = 0.400, *p* = 0.011, Fig. 5B). Of note, there was no correlation between V/Q mismatch improvement and respiratory system compliance at 5 cmH_2_O (Additional file 1: Fig. S2).

### Effects of higher PEEP on respiratory mechanics, gas exchange and hemodynamics

Changes in respiratory mechanics induced by higher PEEP apparently confirmed lung recruitability (Table 3): driving pressure and respiratory system compliance did not worsen despite the large increase in PEEP; the PaO_2_/FiO_2_ ratio and CO_2_ clearance improved (Table 3).

**Table 3:** Effect of positive end expiratory pressure (PEEP) on respiratory mechanics, gas exchange and hemodynamics

	PEEP 5 cmH_2_O *n* = 15	PEEP 15 cmH_2_O *n* = 15	*p* value
*Respiratory mechanics*			
Tidal volume, mL.kg^−1^ PBW	6.9 [6.1–7.6]	6.7 [6.0–7.5]	0.164
RR, min^−1^	22 [16–26]	22 [16–26]	0.334
PEEPtot, cmH_2_O	6 [5–7]	16 [15, 16]	**< 0.001**
Pplat, cmH_2_O	19 [16–21]	28 [26–30]	**< 0.001**
ΔP_RS_, cmH_2_O	12 [10–16]	13 [11–15]	0.508
C_RS_, mL.cmH_2_O^−1^	36 [25–50]	32 [24–42]	0.164
*Gas exchange*			
PaO_2_/FiO_2_, mmHg	125 [69–194]	162 [90–198]	**0.011**
SaO_2_, %	93 [90–96]	95 [93–97]	**0.030**
pH	7.39 [7.34–7.43]	7.38 [7.34–7.42]	0.525
PaCO_2_, mmHg	49.8 [42.0–61.0]	48.0 [42.0–52.1]	0.403
Ventilatory Ratio	2.04 [1.29–2.35]	1.88 [1.33–2.29]	0.248
*Hemodynamics*			
MAP, mmHg	82 [74–98]	76 [70–90]	**0.002**
Pulsed pressure, mmHg	76 [60–85]	62 [56–80]	**0.005**
CVP, mmHg	5 [1–9]	6 [6–13]	**0.007**
HR, bpm	82 [69–99]	81 [65–104]	0.494
ScvO2, %	73 [69–76]	73 [68–76]	0.780

Bold identifies significant differences

*C*_RS_ Respiratory system compliance, *CVP* Central venous pressure, *FiO*_*2*_ Inspired fraction on dioxygen, *HR* Heart rate, *MAP* Mean arterial pressure, *PaCO*_*2*_ Arterial partial pressure on carbon dioxide, *PaO*_*2*_ Arterial partial pressure on dioxygen, *PEEPtot* Total positive end expiratory pressure, *Pplat* Plateau pressure, *ΔP*_*RS*_ Driving respiratory system pressure, *RR* Respiratory rate, *SaO*_*2*_ Arterial saturation on dioxygen, *ScvO*_*2*_ Central venous saturation on dioxygen

PEEP increase induced a moderate decrease in mean and pulsed arterial pressure but it did not affect ScvO_2,_ potentially suggesting stable cardiac output (Table 3).

### Correlation between ventilation distribution and changes in ${{\dot{V}} \mathord{\left/ {\vphantom {{\dot{V}} {\dot{Q}}}} \right. \kern-\nulldelimiterspace} {\dot{Q}}}$ mismatch

The effects of PEEP on ventilation distribution measured by “classical” EIT monitoring were correlated with improved regional ${{\dot{V}} \mathord{\left/ {\vphantom {{\dot{V}} {\dot{Q}}}} \right. \kern-\nulldelimiterspace} {\dot{Q}}}$ mismatch (Additional file 1: Fig. S3A). Larger decrease of tidal volume distending the non-dependent region at higher PEEP was associated with a regional fall in wasted ventilation (Additional file 1: Fig. S3B), whereas increased regional ventilation in the dependent regions led to a decrease in wasted perfusion in the same areas (Additional file 1: Figs. S3B and S4 in the Online Supplement). These results could be useful to predict improvement of ${{\dot{V}} \mathord{\left/ {\vphantom {{\dot{V}} {\dot{Q}}}} \right. \kern-\nulldelimiterspace} {\dot{Q}}}$ mismatch at higher PEEP by standard ventilation monitoring by EIT.

## Discussion

This study describes the effects of higher PEEP on regional ${{\dot{V}} \mathord{\left/ {\vphantom {{\dot{V}} {\dot{Q}}}} \right. \kern-\nulldelimiterspace} {\dot{Q}}}$ mismatch in patients with moderate and severe ARDS. These effects can be summarized as follows: in the non-dependent regions of the lungs, both ventilation and perfusion are redistributed from units with high ${{\dot{V}} \mathord{\left/ {\vphantom {{\dot{V}} {\dot{Q}}}} \right. \kern-\nulldelimiterspace} {\dot{Q}}}$ ratio towards units with more physiological ${{\dot{V}} \mathord{\left/ {\vphantom {{\dot{V}} {\dot{Q}}}} \right. \kern-\nulldelimiterspace} {\dot{Q}}}$ ratio, this leading to a large decrease in wasted ventilation; in the middle lung regions, higher PEEP redistributes perfusion from units with low ${{\dot{V}} \mathord{\left/ {\vphantom {{\dot{V}} {\dot{Q}}}} \right. \kern-\nulldelimiterspace} {\dot{Q}}}$ ratio to units with normal ${{\dot{V}} \mathord{\left/ {\vphantom {{\dot{V}} {\dot{Q}}}} \right. \kern-\nulldelimiterspace} {\dot{Q}}}$ ratio and decreases heterogeneity of the distribution of perfusion, thus ensuring a large decrease in wasted perfusion; in the dependent lung, ${{\dot{V}} \mathord{\left/ {\vphantom {{\dot{V}} {\dot{Q}}}} \right. \kern-\nulldelimiterspace} {\dot{Q}}}$ mismatch improves by minor complementary changes in ventilation and perfusion distribution, yielding lower fraction of wasted perfusion. The only slight worsening of regional ${{\dot{V}} \mathord{\left/ {\vphantom {{\dot{V}} {\dot{Q}}}} \right. \kern-\nulldelimiterspace} {\dot{Q}}}$ mismatch occurs in non-dependent units with minor increase of ventilation reaching units with low ${{\dot{V}} \mathord{\left/ {\vphantom {{\dot{V}} {\dot{Q}}}} \right. \kern-\nulldelimiterspace} {\dot{Q}}}$ and slightly larger fraction of wasted perfusion.

This study provides bedside regional quantification of ${{\dot{V}} \mathord{\left/ {\vphantom {{\dot{V}} {\dot{Q}}}} \right. \kern-\nulldelimiterspace} {\dot{Q}}}$ mismatch in moderate and severe ARDS patients. In the non-dependent regions of the lungs, both ventilation and perfusion were redistributed by higher airway pressure, and this determined a large decrease in wasted ventilation. Previous studies indicated that PEEP can also increase wasted ventilation in the non-dependent lung [18]. Indeed, in isolated lungs, the increase in PEEP induced a decrease in regional perfusion in the non-dependent areas, leading to relative increase in non-perfused and high ${{\dot{V}} \mathord{\left/ {\vphantom {{\dot{V}} {\dot{Q}}}} \right. \kern-\nulldelimiterspace} {\dot{Q}}}$ units [21]. In injured lungs, increased wasted ventilation in the non-dependent lung is mainly related to the excessive ventilation reaching these regions, due to alveolar collapse in the dorsal lung [2, 18]. The effects of PEEP in non-dependent regions might critically depend from its ability to stabilize recruitment vs. simply inflate previously aerated lung regions: the correlation between lower wasted ventilation and the R/I ratio that we disclosed might suggest that, in our patients, the shift in ventilation towards dorsal region due to recruitment stabilized by PEEP coupled with minor redistribution of perfusion led to net reduction of wasted ventilation in the non-dependent lung. Interestingly, in a preliminary study performed in 9 patients with COVID-19 ARDS, Perier et al. observed similar very modest redistribution of perfusion from non-dependent regions at higher PEEP [29].

In the middle and dependent lung areas, ${{\dot{V}} \mathord{\left/ {\vphantom {{\dot{V}} {\dot{Q}}}} \right. \kern-\nulldelimiterspace} {\dot{Q}}}$ mismatch improved mostly through reduction in the amount of wasted perfusion. In a study based on ^13^N washout kinetic evaluated by PET-scan performed in sheep with experimental ARDS, Musch et al. described that the regional fraction of perfusion flowing through shunted areas was inversely correlated with aeration measured by CT-scan [22]. These results and ours might suggest that re-opening of collapsed alveoli in more dependent lung regions may also redirect regional perfusion to newly aerated units, decreasing wasted perfusion. Our findings also echo the results of Borges et al. in piglets with recruitable lung injury: PEEP mostly induced a shift in ventilation towards the dependent regions, whereas perfusion was poorly affected [25]. In that study, higher airway pressure due to larger tidal volume led to a redistribution of perfusion by overdistension and regional increase in pulmonary vascular resistance, ultimately worsening dead space. This suggests that caution might be necessary to generalize our results to all moderate and severe ARDS patients. Indeed, in previous MIGET-based study in unselected patients with ARDS, Ralph et al. described an important individual variability of changes in global ${{\dot{V}} \mathord{\left/ {\vphantom {{\dot{V}} {\dot{Q}}}} \right. \kern-\nulldelimiterspace} {\dot{Q}}}$ mismatch in response to PEEP [30].

Alveolar recruitment already showed potential to decrease the risk of VILI by limiting regional lung strain and atelectrauma [31]: our results on the correlations between improved ${{\dot{V}} \mathord{\left/ {\vphantom {{\dot{V}} {\dot{Q}}}} \right. \kern-\nulldelimiterspace} {\dot{Q}}}$ mismatch and the R/I ratio confirms that application of higher PEEP to recruitable lungs might decrease the risk of VILI through multi-factorial mechanisms, adding protection by reduced wasted perfusion and ventilation to mechanical factors.

Our observations also add to recent study in severely obese ARDS patients, reporting that the application of higher PEEP in patients with high pleural pressure (i.e., a marker of higher recruitability) is not associated with overdistension and it improves respiratory mechanics and gas exchange without impairing hemodynamics [32]. Thus, bedside evaluation of recruitability may be key to identify patients benefiting from a rise in PEEP, and real-time monitoring of improved ${{\dot{V}} \mathord{\left/ {\vphantom {{\dot{V}} {\dot{Q}}}} \right. \kern-\nulldelimiterspace} {\dot{Q}}}$ mismatch may guide selection of personalized PEEP levels or the need of additional step-up approach (e.g., prone position) [32].

Our analysis of ${{\dot{V}} \mathord{\left/ {\vphantom {{\dot{V}} {\dot{Q}}}} \right. \kern-\nulldelimiterspace} {\dot{Q}}}$ mismatch mimics somehow the MIGET method results. However, there are several differences from the original MIGET analysis, which may be important to highlight. EIT detects topographic regional distribution of changes in ventilation and perfusion, whereas MIGET provides a global functional analysis of inert gas exchange with different solubility characteristics [11]. Thus, EIT-based measure of ${{\dot{V}} \mathord{\left/ {\vphantom {{\dot{V}} {\dot{Q}}}} \right. \kern-\nulldelimiterspace} {\dot{Q}}}$ mismatch, in comparison to MIGET, are regional (vs. global) and less prone to confounding effects by extra-pulmonary factors (e.g., hemodynamics or anatomic defects) [33]. EIT also grants narrower ranges of ${{\dot{V}} \mathord{\left/ {\vphantom {{\dot{V}} {\dot{Q}}}} \right. \kern-\nulldelimiterspace} {\dot{Q}}}$ ratios intervals used to build the Gaussian distribution even though the final output in terms of distribution of ventilation and perfusion across ${{\dot{V}} \mathord{\left/ {\vphantom {{\dot{V}} {\dot{Q}}}} \right. \kern-\nulldelimiterspace} {\dot{Q}}}$ ratios was similar [11]. Finally, global ${{\dot{V}} \mathord{\left/ {\vphantom {{\dot{V}} {\dot{Q}}}} \right. \kern-\nulldelimiterspace} {\dot{Q}}}$ mismatch assessed by MIGET represents an average of different regions, failing to detect local triggers for VILI [11, 34], whereas EIT may be more sensitive method to detect increased risk of injury in specific regions.

This study has limitations. First, the sample size was relatively small, and the results should be generalized with caution. However, this was a physiological highly detailed study using a new method to quantify regional ${{\dot{V}} \mathord{\left/ {\vphantom {{\dot{V}} {\dot{Q}}}} \right. \kern-\nulldelimiterspace} {\dot{Q}}}$ mismatch and a sample of 15 patients is similar to previous studies on this topic. Second, we measured lung recruitability by EIT-based calculation of the R/I ratio [35, 36], which may differ from the one based on respiratory system mechanics. However, we chose to focus on the correlation between R/I ratio and ${{\dot{V}} \mathord{\left/ {\vphantom {{\dot{V}} {\dot{Q}}}} \right. \kern-\nulldelimiterspace} {\dot{Q}}}$ mismatch improvement, more than on predefined thresholds, as this approach would have conveyed more relevant information. Third, we didn’t measure cardiac output at lower and higher PEEP levels, thus pixel-level ${{\dot{V}} \mathord{\left/ {\vphantom {{\dot{V}} {\dot{Q}}}} \right. \kern-\nulldelimiterspace} {\dot{Q}}}$ ratios measured by EIT were relative and not absolute. However, in moderate and severe ARDS, minute ventilation is increased due to high dead space and increased CO_2_ production and the difference from increased cardiac output due to sepsis should be minimal, yielding minor difference between absolute and relative ${{\dot{V}} \mathord{\left/ {\vphantom {{\dot{V}} {\dot{Q}}}} \right. \kern-\nulldelimiterspace} {\dot{Q}}}$ratios. Moreover, changes in cardiac output between the 2 PEEP levels might have affected intrapulmonary shunt by mechanisms other than recruitment. The stability of ScvO_2_ and the slight changes in arterial and central venous pressure indirectly suggest stable cardiac output, as expected when higher PEEP is applied to recruitable lungs [32]. Fourth, despite well-defined shared characteristics (infectious etiology, accurate assessment of recruitability, standardized PEEP levels, cross-over randomized design) our population might still have had some intrinsic heterogeneity. Patients with COVID-19 pneumonia, and more “classical” ARDS from intra- and extra-pulmonary origin were enrolled. Nonetheless, we believe that, rather than based on etiology, ARDS should be characterized by precise assessment of physiological characteristics (e.g., recruitability) to correctly apply personalized treatments. Finally, we compared two fixed levels of PEEP (i.e., the lowest to define ARDS and the lowest certainly considered as high in clinical practice) rather than personalized high and low PEEP levels. However, the purpose of this study was to describe mechanisms underlying redistribution of ventilation and perfusion by PEEP rather than fine-tuning of PEEP based on such mechanisms. Thus, we selected 2 levels which, even in more severe patients, would have led to significant perturbations of ARDS pathophysiology.

## Conclusions

In moderate and severe ARDS patients, higher PEEP induces improvements of regional ${{\dot{V}} \mathord{\left/ {\vphantom {{\dot{V}} {\dot{Q}}}} \right. \kern-\nulldelimiterspace} {\dot{Q}}}$ mismatch by different mechanisms across all regions: wasted ventilation decreases in the non-dependent areas while wasted perfusion decreases in the middle and dependent lung. Bedside index of recruitability is correlated with improved ${{\dot{V}} \mathord{\left/ {\vphantom {{\dot{V}} {\dot{Q}}}} \right. \kern-\nulldelimiterspace} {\dot{Q}}}$ mismatch. Personalized matching of higher PEEP with patient’s recruitability might limit the risk of VILI due to wasted regional ventilation and perfusion and, in turn, improve clinical outcomes.

## Supplementary Information

**Additional file 1**. Additional tables and figures.

## Data Availability

The datasets used and/or analysed during the current study are available from the corresponding author on reasonable request.
