# Supplementary material for: Effects of PEEP on regional ventilation-perfusion mismatch in the acute respiratory distress syndrome
Source: Crit Care. 2022 Jul 11;26:211. doi: 10.1186/s13054-022-04085-y (PMC9272883; doi:10.1186/s13054-022-04085-y)

Effects of PEEP on regional ventilation-perfusion mismatch in ARDS patients with recruitable lungs

B. Pavlovsky et al.

**Corresponding author**

Tommaso Mauri

Department of Anesthesia, Critical Care and Emergency,

Fondazione IRCCS Ca’ Granda, Maggiore Policlinico Hospital,

Via Sforza 35, 20122 Milan, Italy

Tel.: +39-0255033237

Email: tommaso.mauri@unimi.it

**Online Supplement**

# Methods

## Study population

Exclusion criteria were: age <18 years old, pregnancy, extra-corporeal respiratory support or contra-indication to EIT (e.g. thoracic wounds and burns).

All investigations were performed according to ethical and transparency standards of the Helsinki’s declaration. The institutional review board approved this study (ref. 311_2021), and informed consent was obtained according to local regulations.

## Study protocol

After enrolment, patients were mechanically ventilated on Volume Controlled Ventilation (VCV) by dedicated ventilator (V500 Infinity ventilator, Dräger, Lubeck, Germany). The following ventilator settings were applied: tidal volume 6-7 mL.kg^-1^ PBW, respiratory rate to target pH of 7.30-7.40 and FiO_2_ to obtain SpO_2_ >94% at clinical PEEP. All patients were positioned in the semi-recumbent position.

An EIT belt connected to a commercial device (Pulmovista, Draeger, Lubeck, Germany), was placed below armpits, between the 3^rd^ and 5^th^ intercostal spaces [S1]. To avoid interferences, the anti-bedsore mattress was turned off during all the study procedure [S1].

Each patient was randomly assigned to a cross-over PEEP strategy: (1) PEEP 5 cmH_2_O and then 15 cmH_2_O, or (2) PEEP 15 cmH_2_O followed by 5 cmH_2_O. Each PEEP level was applied for 30 minutes. Synchronized ventilator and EIT tracings, including airway pressure and flow, were continuously recorded with an acquisition sample rate of 50 Hz for the whole study. Towards the end of each step, respiratory mechanics were assessed by performing tele-inspiratory and tele-expiratory occlusions, arterial and central venous blood gases were obtained. Then, a 20-second end-inspiratory occlusion was performed, during which a 10 ml bolus of 5% NaCl solution was rapidly infused trough a central line to assess lung perfusion. All ventilator and EIT data were downloaded and analyzed offline by commercial and custom-made software. Hemodynamics were collected from the clinical monitor at the end of each study phase, too, including invasive measurements of arterial and central venous blood pressure.

## *Data collection.*

The following clinical characteristics were collected at enrollment: age, sex, body mass index (BMI), relevant comorbidities, SAPS II at ICU admission [S2], SOFA score, number of organs failure (based on the SOFA scoring system) [S3], ARDS etiology, days of intubation, clinical ventilation settings and gas exchange.

Total PEEP (PEEPtot), Plateau pressure (Pplat), driving pressure (ΔP_RS_), respiratory system compliance (C_RS_) were computed offline from ventilation tracings by standard formulas. The recruitment to inflation (R/I) ratio was computed between the two PEEP levels using EIT to quantify the actual change in end expiratory lung volume, as previously described [S4]. The Ventilatory Ratio (VR) was calculated from blood gases and ventilation data by standard formula [S5].

## *Regional analysis of V̇/Q̇ mismatch.*

To enhance the understanding of V̇/Q̇ mismatch distribution, we used a novel approach based upon the regional analysis of ventiation and perfusion. Maps of the pixel-level relative distribution of ventilation and perfusion were analyzed by custom-made dedicated software (MATLAB, MathWorks, Natick, MA, USA). V̇/Q̇ distribution was evaluated at the regional level, by dividing the imaging field into 3 same-size regions of interest (ROI) following a ventro-dorsal gradient, namely non-dependent, middle and dependent ROI [S6]. Each pixel unit was considered as ventilated if its tidal impedance variation reached a value above 10% of the maximal pixel impedance variation across the whole lung image [S7]. The same process was applied to build the perfusion map [S6, S7].

The V̇/Q̇ ratio of each pixel within each region was plotted on a logarithmic scale, allowing a mathematically symmetrical analysis of V̇/Q̇ ratios above and below 1 [S7]. The log(V̇/Q̇) value was rounded to its first decimal on a scale between -1 (corresponding to a V̇/Q̇ ratio of 0.1) and 1 (V̇/Q̇ ratio of 10). Units with log(V̇/Q̇) values <-1 or >1 were classified as non-ventilated perfused and non-perfused ventilated, respectively, and approximated to -1 and 1 value. Twenty-one compartments were obtained, by approximating their log(V̇/Q̇) values to the nearest 1-point decimal number. For example, a unit with log(V̇/Q̇) ratio of -0.86 was approximated to -0.9, while another with log(V̇/Q̇) ratio of 0.32 became 0.3. This operation led to 10 compartments including log(V̇/Q̇) values from -1 to -0.1, a central one with a log(V̇/Q̇) value of 0, and 10 more with log(V̇/Q̇) values from 0.1 to 1. Finally, the distribution curves of ventilation and perfusion based on the log(V̇/Q̇) ratio range were built based on the 21 measured points for each patient in each region at each PEEP level (6 regional curves with 21 points per curve per patient, Figure S1 of the Online Supplement). These distribution curves were fitted with a Gaussian and bi-modal curve [S7] and the one associated with the lowest residual sum of squares (RSS) was retained. Of note, the fitting model robustness was controlled by ensuring that the RSS was below its critical value of 30.14 computed on a Chi^2^ table for 19 degrees of freedom (extreme points corresponding to log(V̇/Q̇) values of -1 and 1 being excluded from the fitting model), to reach a r^2^ value >0.95 and a p-value <0.05 [S7].

## *Quantitative assessment of regional V̇/Q̇ mismatch.*

The shape of the regional ventilation and perfusion over V̇/Q̇ ratio curves was also analyzed to provide their mean distribution in terms of V̇/Q̇ ratio (Mean V and Mean Q) and their skewness by logarithmic standard derivation (logSD_V_ and logSD_Q_), as follows [S8]:

1. logMean V̇ = $\frac{\sum_{i=1}^{n} (\log\left( \frac{V}{Q} \right)i*Vti)}{\sum_{i=1}^{n} Vti}$ , then Mean V̇ = 10^logMeanV̇^

where n is the number of pixels in the functional EIT image within each ROI.

1. logMean Q̇ = $\frac{\sum_{i=1}^{n} (\log\left( \frac{V}{Q} \right)i*Qi)}{\sum_{i=1}^{n} Qi}$ , then Mean Q̇ = 10^logMeanQ̇^

where n is the number of pixels in the functional EIT image within each ROI.

1. logSD_V̇_ = $\frac{\sum_{i=1}^{n} [\log\left( \frac{V}{Q} \right)i-logMeanV^{2}]*Vti)}{\sum_{i=1}^{n} Vti}$

where n is the number of pixels in the functional EIT image within each ROI.

1. LogSD_Q̇_ = $\frac{\sum_{i=1}^{n} [\log\left( \frac{V}{Q} \right)i-logMeanQ^{2}]*Qi)}{\sum_{i=1}^{n} Qi}$

where n is the number of pixels in the functional EIT image within each ROI.

Finally, precise assessment of regional wasted perfusion and wasted ventilation was calculated by the equations (5) and (6).

1. $Wasted perfusion= \sum_{i=1}^{n} \left( {\log\left( \dot{\frac{\dot{V}}{Q}} \right)}_{i}*Q_{i} \right)$

where n is the number of pixels in the functional EIT image within each ROI, including only units with V̇/Q̇ ratio <1

1. $Wasted ventilation= \sum_{i=1}^{n} \left( {\log\left( \dot{\frac{\dot{V}}{Q}} \right)}_{i}*{Vt}_{i} \right)$,

where n is the number of pixels in the functional EIT image within each ROI, including only units with V̇/Q̇ ratio >1

For both equations, the absolute value was taken in account (especially for the wasted perfusion, to avoid a negative result).

# Supplement References

1. Frerichs I, Amato MB, van Kaam AH, Tingay DG, Zhao Z, Grychtol B, Bodenstein M, Gagnon H, Böhm SH, Teschner E, Stenqvist O, Mauri T, Torsani V, Camporota L, Schibler A, Wolf GK, Gommers D, Leonhardt S, Adler A; TREND study group. Chest electrical impedance tomography examination, data analysis, terminology, clinical use and recommendations: consensus statement of the TRanslational EIT developmeNt stuDy group. Thorax. 2017;72(1):83-93.
2. Le Gall JR, Lemeshow S, Saulnier F. A new simplified acute physiology score (SAPS II) based on a European/North American multicenter study. JAMA. 1993;270:2957–63.
3. Vincent JL, Moreno R, Takala J, Willatts S, De Mendonça A, Bruining H, Reinhart CK, Suter PM, Thijs LG. The SOFA (Sepsis-related Organ Failure Assessment) score to describe organ dysfunction/failure. On behalf of the Working Group on Sepsis-Related Problems of the European Society of Intensive Care Medicine. Intensive Care Med. 1996;22(7):707-10.
4. Mauri T, Spinelli E, Scotti E, Colussi G, Basile MC, Crotti S, Tubiolo D, Tagliabue P, Zanella A, Grasselli G, Pesenti A. Potential for Lung Recruitment and Ventilation-Perfusion Mismatch in Patients With the Acute Respiratory Distress Syndrome From Coronavirus Disease 2019. Crit Care Med. 2020;48(8):1129-1134.
5. Sinha P, Calfee CS, Beitler JR, Soni N, Ho K, Matthay MA, Kallet RH. Physiologic Analysis and Clinical Performance of the Ventilatory Ratio in Acute Respiratory Distress Syndrome. Am J Respir Crit Care Med. 2019;199(3):333-341.
6. Borges JB, Cronin JN, Crockett DC, Hedenstierna G, Larsson A, Formenti F. Real-time effects of PEEP and tidal volume on regional ventilation and perfusion in experimental lung injury. Intensive Care Med Exp. 2020;8(1):10.
7. Wagner PD, Saltzman HA, West JB. Measurement of continuous distributions of ventilation-perfusion ratios: theory. J Appl Physiol. 1974;36(5):588-99.
8. Rodriguez-Roisin R, Roca J, Agusti AG, Mastai R, Wagner PD, Bosch J. Gas exchange and pulmonary vascular reactivity in patients with liver cirrhosis. Am Rev Respir Dis. 1987;135(5):1085-92.

# Additional Table

| **V̇ and Q̇ distribution** | **PEEP 5 cmH_2_O**  **n = 15** | **PEEP 15 cmH_2_O**  **n = 15** | **p value** |
| --- | --- | --- | --- |
|  |  |  |  |
| **Non-dependent region** |  |  |  |
| **V̇, %** | 51 [39-62] | 38 [35-46] | **<.001** |
| **Q̇, %** | 29 [20-39] | 28 [22-32] | .309 |
|  |  |  |  |
| **Middle region** |  |  |  |
| **V̇, %** | 44 [33-52] | 49 [41-58] | **<.001** |
| **Q̇, %** | 49 [43-55] | 51 [47-57] | .087 |
|  |  |  |  |
| **Dependent region** |  |  |  |
| **V̇, %** | 9 [4-13] | 10 [6-19] | **.006** |
| **Q̇, %** | 14 [9-21] | 16 [11-23] | .855 |
|  |  |  |  |

**Table S1. Fractions of ventilation and perfusion at the 2 PEEP levels in the 3 regions of interest.**

| **V̇ and Q̇ distribution** | **PEEP 15 cmH_2_O** | | **p value** | **PEEP 5 cmH_2_O** | | **p value** |
| --- | --- | --- | --- | --- | --- | --- |
|  | **5-15 order**  **n = 8** | **15-5 order**  **n = 7** |  | **5-15 order**  **n = 8** | **15-5 order**  **n = 7** |  |
|  |  |  |  |  |  |  |
| **Non-dependent region** |  |  |  |  |  |  |
| **V̇, %** | 39 [36-46] | 38 [26-46] | .410 | 53 [43-61] | 46 [37-64] | .613 |
| **Q̇, %** | 28 [23-31] | 31 [21-33] | .380 | 29 [25-35] | 38 [13-43] | .514 |
|  |  |  |  |  |  |  |
| **Middle region** |  |  |  |  |  |  |
| **V̇, %** | 49 [42-54] | 53 [39-65] | .779 | 40 [34-46] | 49 [24-60] | .759 |
| **Q̇, %** | 54 [48-59] | 50 [44-54] | .336 | 51 [49-56] | 43 [42-50] | .051 |
|  |  |  |  |  |  |  |
| **Dependent region** |  |  |  |  |  |  |
| **V̇, %** | 8 [6-18] | 12 [8-23] | .346 | 8 [3-13] | 9 [5-16] | .349 |
| **Q̇, %** | 14 [9-23] | 16 [11-23] | .755 | 16 [8-21] | 14 [10-26] | .613 |
|  |  |  |  |  |  |  |

**Table S2. Fractions of ventilation and perfusion at the 2 PEEP levels in the 3 regions of interest, according to randomization order groups.**

# Additional Figures

**Figure S1. Individual patient data of the distribution of regional Ventilation (blue curves) and Perfusion (red curves) across all V̇/Q̇ ratios.**

ND: non-dependent part of the lungs, M: middle part of the lungs, D: dependent part of the lungs.

**Figure S2. Correlations between respiratory system compliance (C_RS_) at PEEP 5 cmH_2_O, and the improvement in Wasted Ventilation (A) and Wasted Perfusion (B) between PEEP 5 and 15 cmH_2_O.**

**Figure S3. The decrease in wasted ventilation in the non-dependent lung was correlated with the decrease in regional ventilation between PEEP 5 and 15 cmH_2_O (Panel A).** **In the dependent lung, reduced wasted perfusion was correlated with the increase in regional ventilation between PEEP 5 and 15 cmH_2_O (Panel B).**

ND: non-dependent part of the lungs, D: dependent part of the lungs.

**Figure S4. In the middle lung region, reduced wasted perfusion was correlated with the increase in regional ventilation between PEEP 5 and 15 cmH_2_O.**


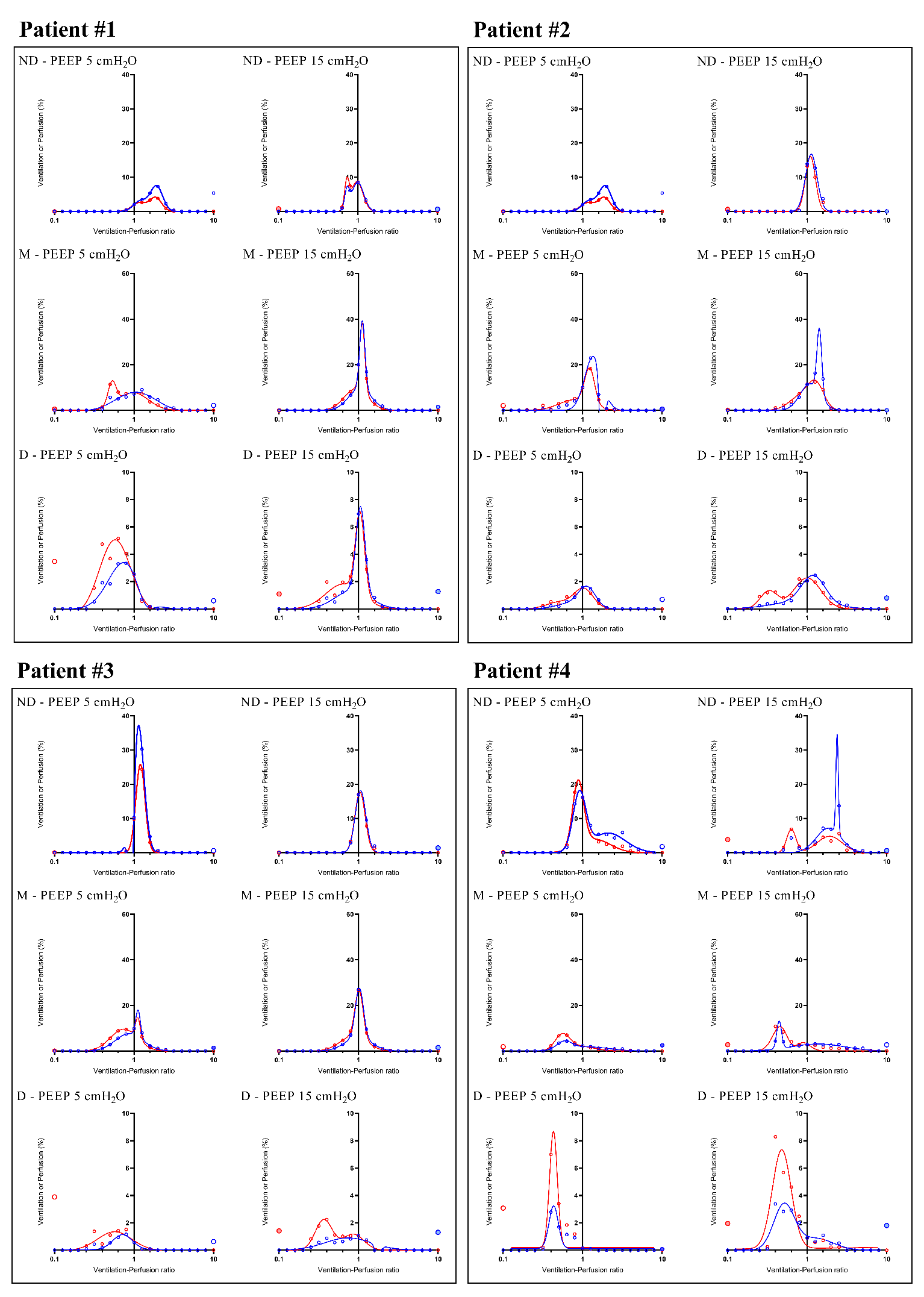


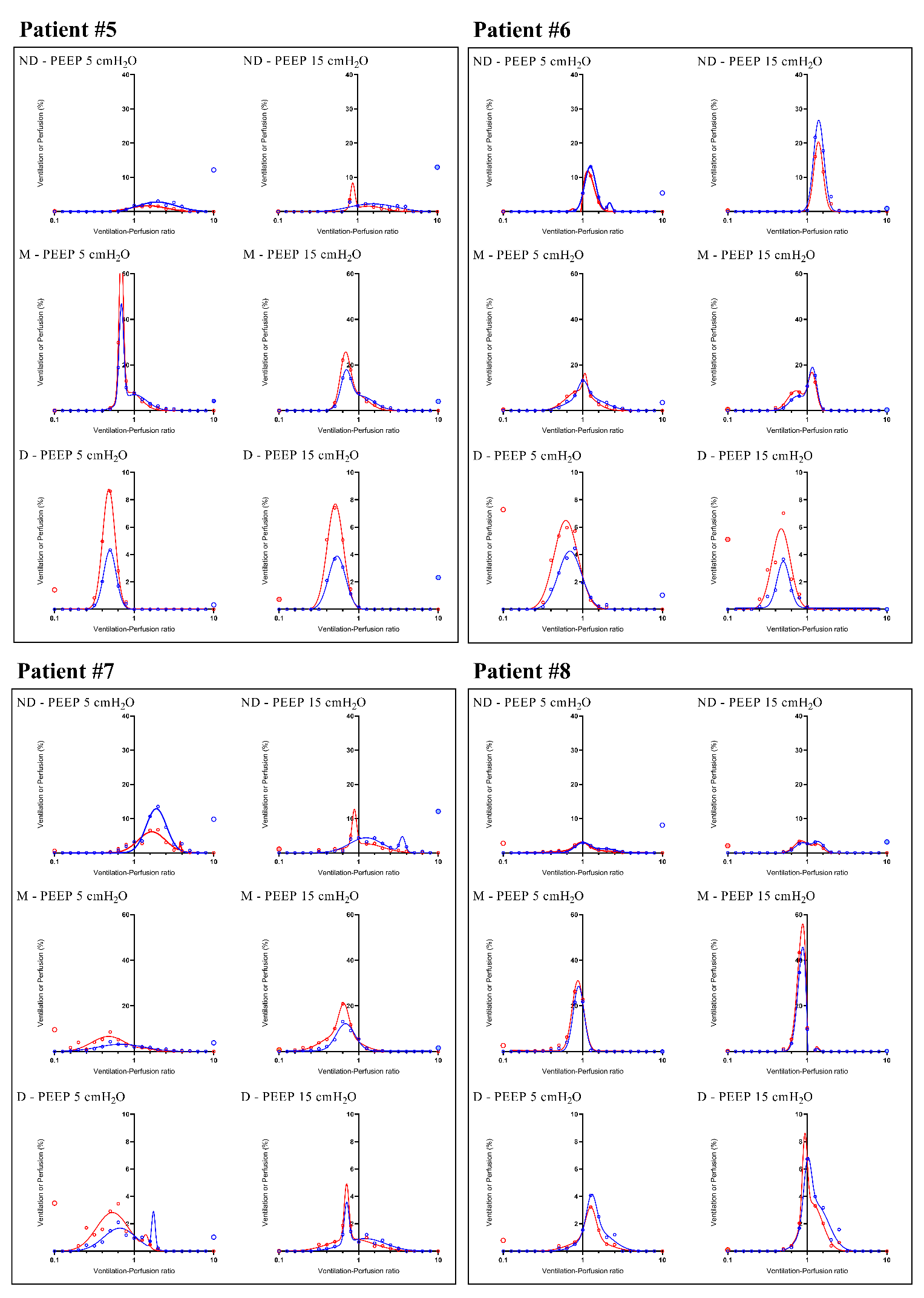


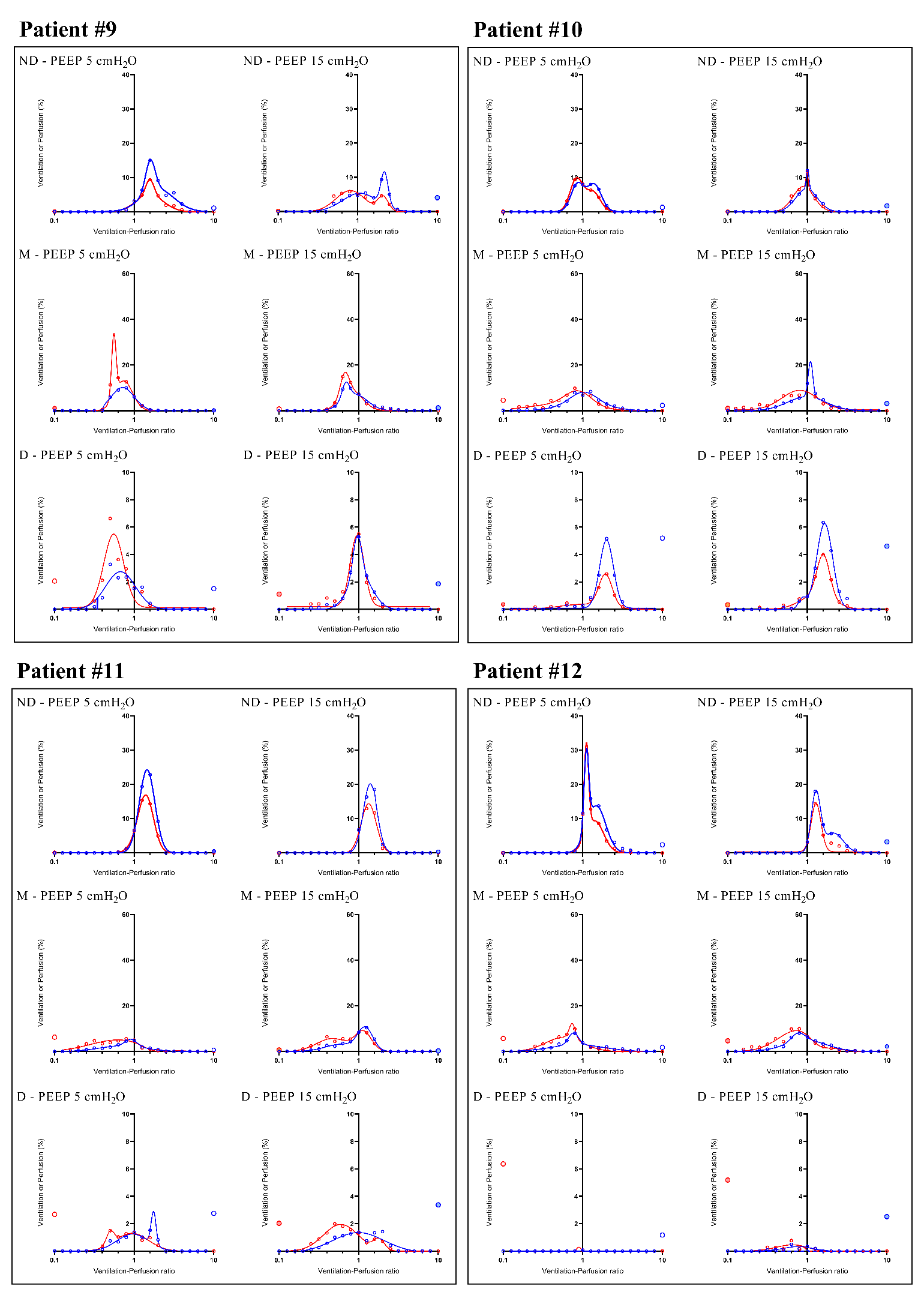


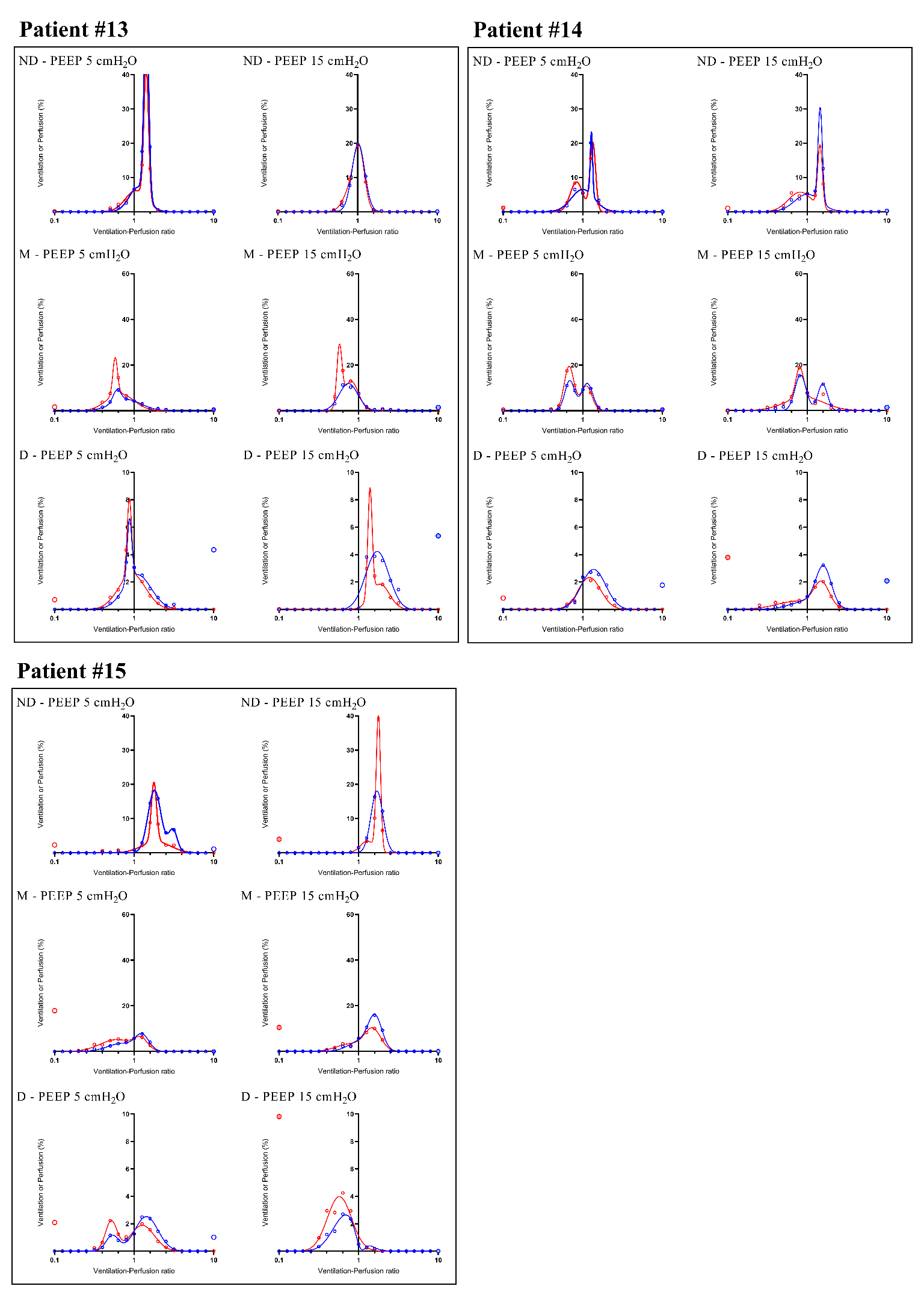


**Figure S2**

**
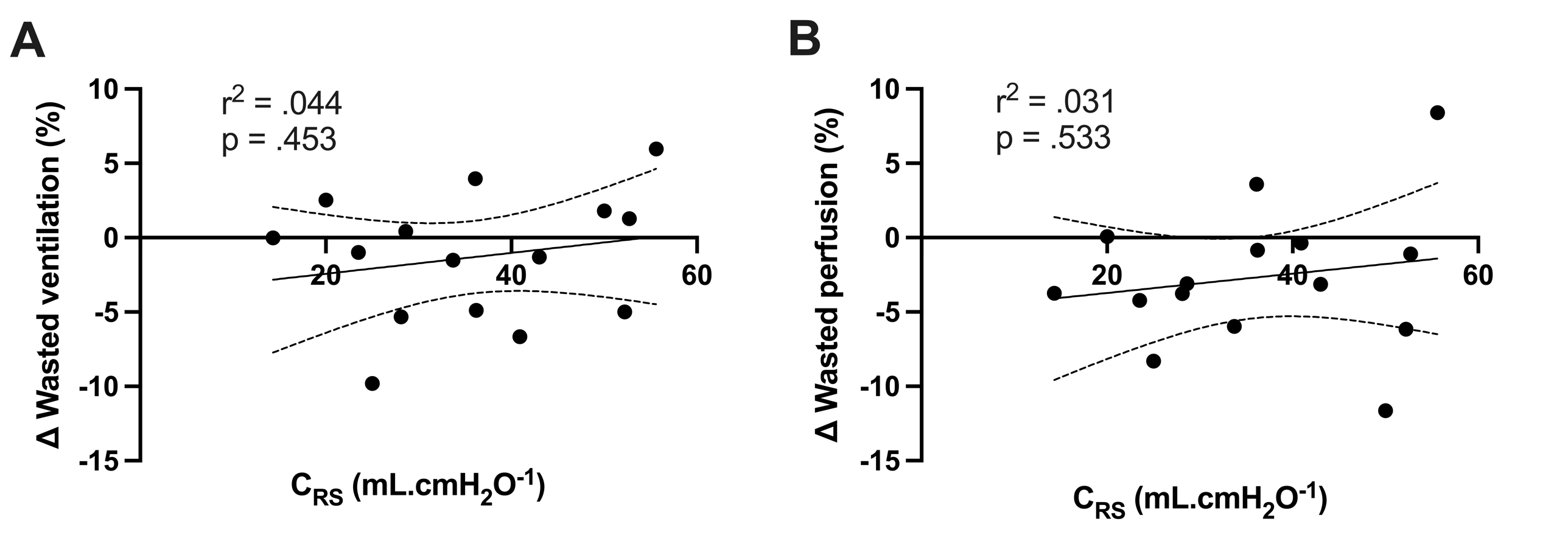
**

**Figure S3**


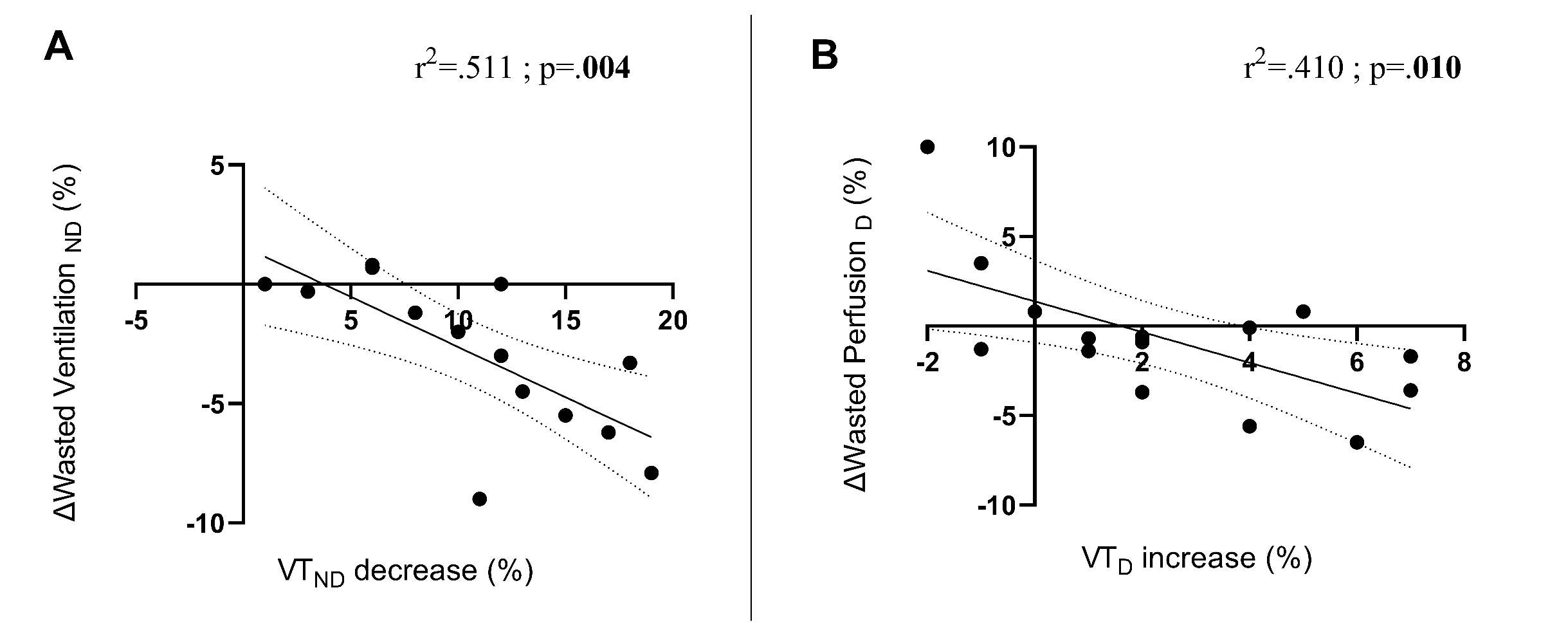


**Figure S4**


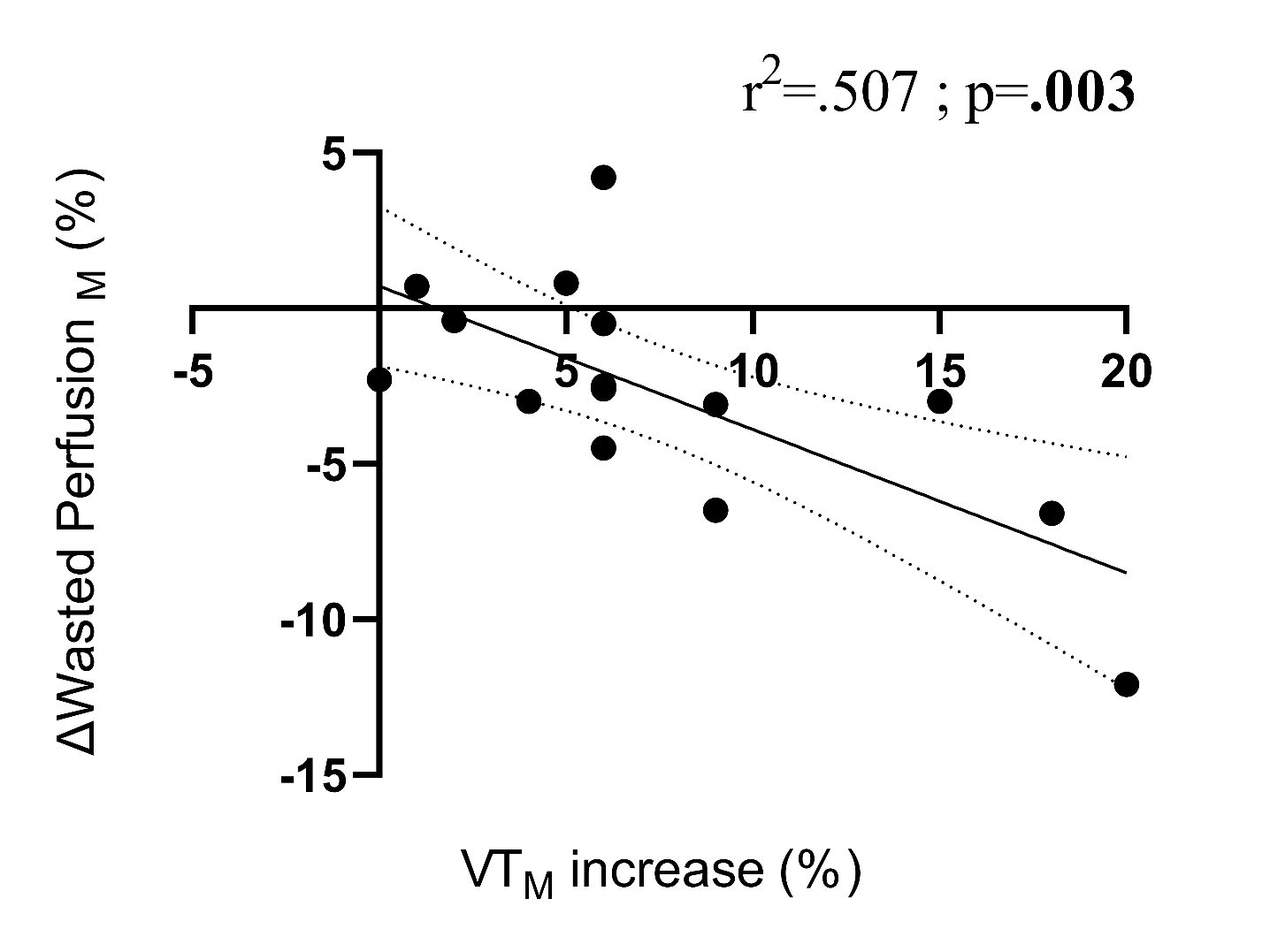

Supplement: Supplementary file 1 — Additional file 1. Additional tables and figures. [file 13054_2022_4085_MOESM1_ESM.docx]
